# MINSTED nanoscopy enters the Ångström localization range

**DOI:** 10.1038/s41587-022-01519-4

**Published:** 2022-11-07

**Authors:** Michael Weber, Henrik von der Emde, Marcel Leutenegger, Philip Gunkel, Sivakumar Sambandan, Taukeer A. Khan, Jan Keller-Findeisen, Volker C. Cordes, Stefan W. Hell

**Affiliations:** 1 Department of NanoBiophotonics, Max Planck Institute for Multidisciplinary Sciences, Göttingen, Germany; 2 Department of Cellular Logistics, Max Planck Institute for Multidisciplinary Sciences, Göttingen, Germany; 3 Synaptic Metal Ion Dynamics and Signaling, Max Planck Institute for Multidisciplinary Sciences, Göttingen, Germany; 4 Laboratory of Neurobiology, Max Planck Institute for Multidisciplinary Sciences, Göttingen, Germany; 5 Department of Optical Nanoscopy, Max Planck Institute for Medical Research, Heidelberg, Germany

**Keywords:** Super-resolution microscopy, Super-resolution microscopy

## Abstract

Super-resolution techniques have achieved localization precisions in the nanometer regime. Here we report all-optical, room temperature localization of fluorophores with precision in the Ångström range. We built on the concept of MINSTED nanoscopy where precision is increased by encircling the fluorophore with the low-intensity central region of a stimulated emission depletion (STED) donut beam while constantly increasing the absolute donut power. By blue-shifting the STED beam and separating fluorophores by on/off switching, individual fluorophores bound to a DNA strand are localized with *σ* = 4.7 Å, corresponding to a fraction of the fluorophore size, with only 2,000 detected photons. MINSTED fluorescence nanoscopy with single-digit nanometer resolution is exemplified by imaging nuclear pore complexes and the distribution of nuclear lamin in mammalian cells labeled by transient DNA hybridization. Because our experiments yield a localization precision *σ* = 2.3 Å, estimated for 10,000 detected photons, we anticipate that MINSTED will open up new areas of application in the study of macromolecular complexes in cells.

## Abstract (web summary)

Sub-nanometer localization precision is achieved with all-optical microscopy.

## Main

Since the 1970s, fluorescence microscopy has been indispensable for studying the distribution of biomolecules in cells. At the turn of this century, STED microscopy^1^ broke the diffraction barrier that imposed an apparently unsurmountable physical limit on optical resolution, opening up the imaging of cells at the tens of nanometers scale. This transformation has become possible by relying on the on/off switching of the ability of fluorophores to fluoresce. The recently introduced MINFLUX^2^ and MINSTED^3^ nanoscopy added another factor of ten, thus finally reaching a resolution at the scale of the fluorescence labels.

MINFLUX and MINSTED uniquely combine the specific strongpoints of STED and the method called PALM/STORM^4^. Like the latter, they switch the fluorescence ability individually per fluorophore, ensuring the finest possible discrimination of neighboring fluorophores. However, unlike in PALM/STORM, where the stochastic, initially unknown position of the fluorophore is derived from the diffraction spot of fluorescence detections emerging on a camera, in MINFLUX and MINSTED the individual fluorophores are localized with a movable reference point in the sample that is usually defined by the intensity minimum of a donut-shaped beam. By moving this donut minimum closer to the position of the fluorophore during the localization process, MINFLUX and MINSTED increase the information gain per detected photon so that precisions of σ ≈ 1–2 nm are routinely attained with only 200–1,000 photons on single fluorophores. Clearly, once 3*σ* < 1 nm and the molecular construct linking the fluorophores to the target biomolecules is controlled, structural biology type of studies inside cells should become viable using optical microscopes.

A major factor limiting the attainable precision is background—that is, photon detections not stemming from the target fluorophore. Initial experiments have shown that MINSTED has an advantage over MINFLUX in this regard, because its donut-shaped STED beam is designed to suppress fluorescence. This is contrary to MINFLUX where the donut elicits fluorescence in an area that is about three times larger than in a standard confocal microscope. Besides, building on a STED microscope that inherently offers resolution tuning by changing the donut power, a MINSTED setup can readily accommodate a resolution ranging from the diffraction limit to the molecular scale. Nonetheless, our initial MINSTED study revealed that subtle heating by the STED beam, probably of the sample and the lens immersion oil, limits the precision to *σ* > 1 nm. This is because the popular STED beam of wavelength *λ*_STED_ = 775 nm entails a several orders of magnitude higher average power than what is typically used in confocal and MINFLUX microscopy. By and large, if cryogenic temperatures^5^ are not acceptable, finding a solution that further reduces *σ* is exceedingly challenging.

Here we report MINSTED attaining all-optical fluorophore localization with precisions in the Ångström range. Corresponding to a fraction of the fluorophore size, these precisions are attained at room temperature using a STED microscope. Individual fluorophores on a DNA strand are localized with *σ* = 4.7 Å, measured by dividing localizations into overlapping blocks of 2,000 photons from single emission traces. For the total of 10,000 photons actually detected in the traces, a precision *σ* = 2.3 Å is estimated. MINSTED fluorescence nanoscopy with nanometer resolution is exemplified by imaging nuclear pore complexes in mammalian cells labeled by DNA hybridization as in the method called DNA PAINT^6,7^. Similar resolution is obtained in MINSTED images of the distribution of synaptic proteins in rat hippocampal neurons. These advancements have become possible because, unlike standard STED, MINSTED nanoscopy operates with just a single on-state fluorophore at a time, whereas all other fluorophores in the focal region are off. Moreover, having just a single active fluorophore enables a more effective implementation of STED to the benefit of the MINSTED concept.

## Results

**Fig. 1:**
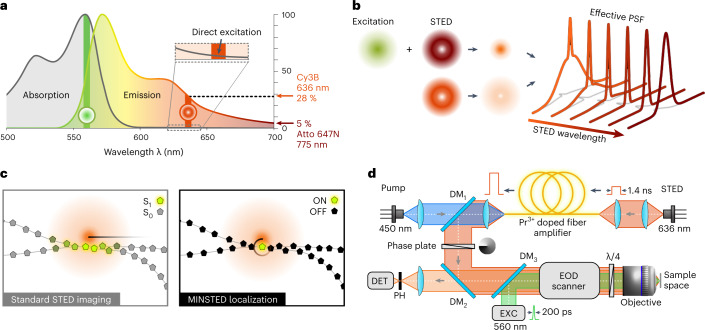
Blue-shifted MINSTED. **a**, Qualitative fluorescence and absorption spectra of the fluorophore Cy3B, including our selection of wavelength for excitation (560 nm, green) and de-excitation by stimulated emission (636 nm, red). Reaching well into the emission peak, the cross-section for stimulated emission amounts to 28% of its global maximum, at the expense of slight ‘direct’ excitation of ground state Cy3B fluorophores by the donut-shaped STED beam (inset). **b**, Blue-shifting the wavelength of the donut (lower donut has shorter wavelength) for a given power sharpens the central peak of the effective PSF of the STED microscope but gives rise to a pedestal. **c**, The pedestal leads to weak fluorescence from bystander fluorophores, thus compromising the contrast in standard STED imaging (left). Because only one fluorophore is active in MINSTED, the pedestal is ineffectual (right), meaning that the benefits of the blue-shifted STED wavelength can be exploited. **d**, Schematic of the MINSTED setup: originating from a 636-nm emitting laser diode, the STED 1.4-ns pulses are amplified by a Pr^3+^ doped fiber pumped with 450-nm laser diode, deflected by a dichroic mirror (DM1), converted into a donut by a phase plate and aligned with a laser emitting 200-ps pulses for excitation at 560 nm. The co-aligned beams are steered in the focal plane of the objective lens by an EOD, whereas the quarter-wave plate (λ/4) ensures circular polarization. Fluorescence collected from the sample is de-scanned, spatially filtered by a pinhole (PH) and detected.

The physics behind our study can be outlined as follows (Fig. 1). In virtually all STED microscopes, including in our initial MINSTED implementation, *λ*_STED_ is tuned to the very red edge of the fluorescence spectrum. For red-orange emitting fluorophores, the popular near-infrared *λ*_STED_ = 775 nm is typically chosen. The reason is that, at room temperature, the excitation spectrum of most fluorophores extends deeply into the emission peak. STED donuts with shorter *λ*_STED_, therefore, tend to ‘directly’ excite many bystander fluorophores in the anti-Stokes mode, overall producing substantial fluorescence in the donut region. This fluorescence consequently compromises the on/off contrast needed for fluorophore separation^8^ (Fig. 1b,c). As the fluorophore cross-section *ς* for stimulated emission scales with the emission spectrum, shifting *λ*_STED_ far out to the red edge decreases *ς* and, thus, the STED efficiency per unit STED beam power. Compensating the decrease in *ς* with increasing power clearly has (thermal load) limits that become apparent when localizing on the finest scale.

However, when the on/off contrast is provided by a process other than STED, as is the case when switching single fluorophores between active and inactive states, *λ*_STED_ can be tuned closer to the emission maximum so that *ς* becomes larger. The background due to ‘direct’ excitation by the STED donut beam is not of concern in this case, because all fluorophores, apart from the one to be localized, are inactive (Fig. 1c). The larger *ς* enables a lower STED beam power so that prohibitive heating can be avoided and pulsed diode lasers can be used (Fig. 1d).

**Fig. 2:**
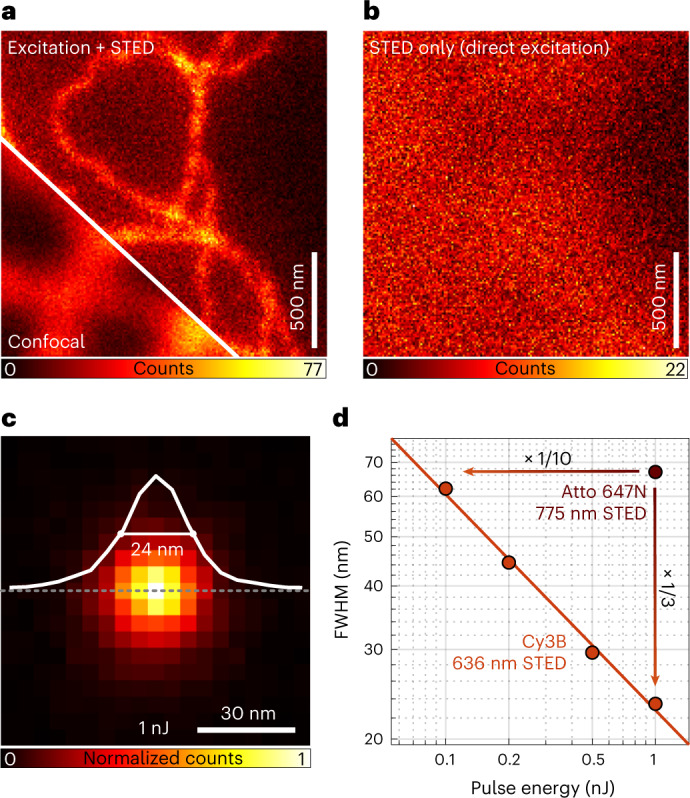
Contrast and resolution of blue-shifted STED. **a**, Confocal and STED comparison images of cellular vimentin immunolabeled with Cy3B using 636-nm wavelength for STED. Note the haze around the vimentin fiber images due to the E-PSF pedestal. **b**, Corresponding fluorescence image produced by ‘direct’ excitation with the STED beam. In both **a** and **b**, a STED pulse energy of 0.5 nJ was applied. **c**, E-PSF with central profile and 24-nm FWHM at STED pulse energy *E* = 1 nJ, measured with immobilized single Cy3B molecules. **d**, FWHM of E-PSF as a function of *E*; FWHM measured with standard 775-nm STED beam on Atto 647N molecules is displayed for comparison.

Blue-shifting *λ*_STED_ also changes the effective point spread function (E-PSF) of the optical setup (Fig. 1b). Being the product of the normalized probability for excitation at *λ*_EXC_ (and *λ*_STED_) and that for de-excitation at *λ*_STED_, the E-PSF represents the probability of a fluorophore to emit at a certain coordinate in the focal region. Generally, the full-width at half-maximum (FWHM) of the E-PSF becomes narrower with increasing *ς* and donut intensity. On the other hand, the blue-shifted *λ*_STED_ causes a pedestal due to ‘direct’ excitation by the donut (Fig. 1b). Although this pedestal compromises bulk STED imaging (Fig. 2a,b), it is not of concern when addressing solitary emitters.

For the fluorophore Cy3B with emission peaking at ≈570 nm, we implemented *λ*_STED_ = 636 nm so that *ς* reached 28% of its global maximum. This should be contrasted with the ≈5% obtained when applying the popular *λ*_STED_ = 775 nm to red emitting fluorophores like Atto647N. The co-aligned *λ*_EXC_ = 560 nm excitation and STED beams, having electronically synchronized pulses of 200 ps and 1.4-ns duration, respectively, were focused into the sample by an oil immersion objective of 1.4 numerical aperture. Thus, an E-PSF of 24-nm lateral FWHM was gained at a STED pulse energy of 1 nJ (Fig. 2c,d). In comparison, pulses of ≈10 nJ would be needed to attain the same FWHM with Atto 647N at *λ*_STED_ = 775 nm. At the used 10-MHz repetition rate, this blue shift entailed a reduction of the average STED beam power from 100 mW to 10 mW, thus substantially lowering the thermal load. Note that tuning the FWHM continually from confocal down to a minimal FWHM is an integral part of each MINSTED fluorophore localization.

**Fig. 3:**
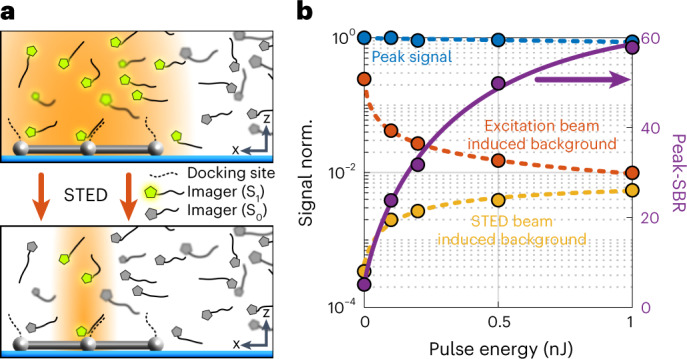
Combining STED with on/off switching and labeling by DNA hybridization. **a**, Fluorophores (pentagons in gray and highlighted in green when able to fluoresce) attached to single-stranded DNA diffusing in solution, sporadically binding to molecular targets having complementary DNA strands; here the target is a DNA origami represented by gray spheres and sticks. The region in which fluorescence is possible (that is, E-PSF region) is shown in orange for the confocal case (top panel) and the STED case (lower panel). Suppression of the fluorescence of the quickly diffusing fluorophores by STED increases the ratio between the fluorescence signal of bound (on) and diffusing (off) fluorophores. The increased on/off ratio enhances the detection of single bound fluorophores. Conversely, it can be used to increase the concentration of diffusing fluorophores so to increase the imaging speed. **b**, Peak fluorescence from single DNA-bound Cy3B fluorophores (blue), fluorescence from diffusing fluorophores with excitation and STED, subtracted the STED-only signal. This is considered as the signal produced from the center peak of the E-PSF by the diffusing fluorophores (red), and STED beam induced fluorescence of the diffusing fluorophores (orange) as a function of the STED pulse energy *E*. The SBR increases by a factor >10 over that of confocal microscopy due to application of *E* = 1-nJ STED pulses.

For separation by on/off switching, we first opted for the mechanism implemented in the method called DNA PAINT: the transient binding of fluorophores to the biomolecular targets of interest via DNA hybridization. It is ‘on’ when, bound to a target, the fluorophore emits from the same coordinate. Conversely, the fluorophore is ‘off’ when it diffuses in the surrounding medium, generating just a weak background (Fig. 3). This on/off modulation by binding and diffusion allowed us to avoid photo-activatable and photo-switchable fluorophores and employ regular fluorophores instead, specifically Cy3B. Thus, PAINT allowed us to use dyes that are highly suitable for STED at our preferred wavelength. The on/off modulation by DNA PAINT also made it possible to measure individual binding sites multiple times, which facilitated the statistical analysis of the localization precision on single binding sites. In general, the combination of STED with labeling by DNA hybridization is highly synergistic, because when localizing a bound fluorophore, the STED donut suppresses the background from the diffusing fluorophores. The use of STED simply increases the DNA labeling contrast by adding another off-switching mechanism. Likewise, this amplified off-switching facilitates employing higher concentrations of diffusing labels compared to standard high-resolution DNA PAINT applications, so that the imaging can be accelerated^9^.

**Fig. 4:**
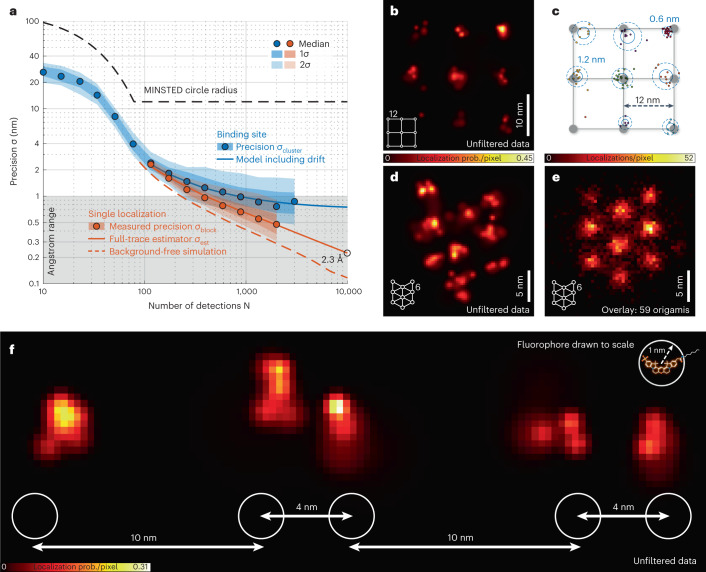
Localization precision and resolution in MINSTED nanoscopy. **a**, Localization precision (points: median; shaded areas: ±1 and ±2 standard deviations) measured from many consecutive binding events on clustered binding sites (blue) on DNA origami grids of 12-nm periodicity and for each binding event individually (red). Blue points and shades are displayed only if computed from at least ten clusters. The red solid line shows the estimated localization precision for the individual events; resulting from that, instabilities are considered to reconstruct the cluster data (blue solid line). Simulated localizations without background are shown as the red dashed line. **b**, MINSTED image of rectangular binding site pattern of 12-nm periodicity and the pertinent localization distribution in **c**. Each localization is represented by its estimated position. Blue circles correspond to 1 (solid) and 2 (dashed) standard deviations of the estimated binding site position. The cluster identity is color-coded. **d**, MINSTED image of 3 × 3 hexagonal DNA origami with internal distances of 6 nm. **e**, Overlay of 59 MINSTED images of the origami pattern from **d**, completely resolving the periodically arranged bindings of 6-nm mutual distance. The data were filtered according to Supplementary Table 1. **f**, Binding sites of 4-nm distance are fully resolved by MINSTED; sketch of the underlying origami design is shown below. The circles of 2-nm diameter represent the extent of the Cy3B molecules whose structure is drawn to scale (upper-right corner) to highlight the relationship between the localization precision and the fluorophore size.

To quantify our blue-shifted MINSTED localization and nanoscopy, we carried out measurements with rectangular DNA origami arrays offering 3 × 3 binding sites for individual fluorophores at 12-nm periodic distance. The concentration of diffusing Cy3B fluorophores was chosen such that only one fluorophore docked to the grid points within the focal region at a time, whereas the other fluorophores diffused freely in solution (Figs. 3b and 4a–c). First, we verified the gain in signal-to-background ratio (SBR) with increasing STED pulse energy *E* (Fig. 3b). To this end, we raster-scanned a 2 µm × 2 µm field of view, applying an excitation average power of 1.5 µW. The peak fluorescence rendered by individually bound fluorophores was extracted from the fluorescence maxima in the resulting image, whereas the background was derived from the mean fluorescence signal per pixel. Recordings with the excitation beam turned off allowed us to quantify the ‘direct’ excitation by the STED beam. We found that the dominant background component was indeed due to the excitation beam inducing fluorescence from diffusing fluorophores. However, this component rapidly dropped with increasing STED pulse energy *E*, because the donut confined the region where fluorescence was allowed. ‘Direct’ excitation by the STED beam increased with *E* but remained acceptable (Fig. 3b). Altogether, the resulting SBR = 60 at 1 nJ was >10 times higher than the SBR obtained by standard confocal microscopy (*E* = 0) and also not sacrificed as in the typical last localization steps of MINFLUX, setting the ground for high-precision localization.

Localization of a fluorophore docking onto a binding site was accomplished by applying the previously detailed MINSTED procedure^3^. In brief, we scanned the co-aligned excitation and STED beams circularly around the fluorophore while continuously increasing *E* so that the fluorophore always experienced the steep edge of the E-PSF. In this constellation, the probability of a fluorophore to fluoresce is roughly equal to its probability to undergo de-excitation through stimulated emission; in other words, de-excitation is not ‘saturated’. By adjusting the scanning radius *R* to half of the FWHM ($R_i = {{{\mathrm{FWHM}}}}_i/2$) of the E-PSF, this condition was kept throughout the measurement for every detected photon. Thus, the detection probability became indicative of the fluorophore’s position with respect to the predetermined position of the donut zero. By the same token, the fluorophore always experienced the same low intensity of the STED beam, irrespective of the STED beam power actually applied. Although we started out with pulse energy *E* = 0, meaning a confocal E-PSF, *E* was continually increased for every detected photon *i*; the FWHM_*i*_ of the E-PSF thus decreased accordingly. At the same time, the circle center was shifted toward the direction of each detection by 0.15 *R*_*i*_ and was, therefore, tightly linked with the FWHM_*i*_ of the E-PSF. After reaching *E* = 1 nJ and the minimal FWHM_min_ of the E-PSF, as well as the minimal radius *R*_min_ = 24 nm / 2 = 12 nm, the E-PSF and *R*_*i*_ were left constant. This was typically the case for *i* = *N*_*c*_ ≈ 80 detected photons. Afterwards, the circle center position was still updated until the localization ended. The resulting measurement is a series of circle center positions (*x*_*j,i*_,*y*_*j,i*_) representing fluorophore coordinate updates until *i* reaches *L*, the total number of detected photons in each localization trace. The index *j* refers to different localizations—that is, fluorophores. We recorded up to *j* = 991 localizations in total, spread over ≈144 binding sites. This set of center positions allowed us to quantify the MINSTED localization precision.

Of all localizations, 90% featured a standard deviation $\sigma \left( {x_{j,i}} \right)_{N_c \le i \le L}$ of the center positions below *σ*_c_ = 4.2 nm in both *x* direction and *y* direction (Supplementary Table 1). In other words, once the minimal FWHM at *i* = *N*_c_ detected photons was reached, the center positions converged within an area defined by *σ*_c_. The remaining localizations were thought to be compromised by the binding of a second fluorophore or other sources of background and were filtered out. The vast majority of localizations could be assigned to specific binding sites of the grid patterns just by grouping the localizations in clusters maximally covering 10 nm in diameter. Clusters with fewer than five localizations were discarded, which resulted in a total number of 59 clusters. Provided the binding sites are firm, the spread of the multiple localizations per cluster yields the localization precision.

The precision resulting from repeated localizations of the same binding site, $\sigma _{{{{\mathrm{cluster}}}}}\left( N \right),$ was computed as a function of the photon number *N*. For $1 < N \le N_{{{\mathrm{c}}}}$, the precision $\sigma _{{{{\mathrm{cluster}}}}}(N)$ was established as the standard deviation $\sqrt {\sigma \left( {x_{j,N}} \right)_{j \in K}\sigma \left( {y_{j,N}} \right)_{j \in K}}$ of the center positions $\left( {x_{j,N},y_{j,N}} \right)$ in each cluster of index *K*. Once the minimum FWHM had been reached—that is, for $N > N_{{{\mathrm{c}}}}$—the fluorophore position was equated with the average of the measured center positions $\left( {\bar x_{j,N},\bar y_{j,N}} \right) = \left\langle\left( {x_{j,i},y_{j,i}} \right)\right\rangle_{N_{{{\mathrm{c}}}} \le i \le N}$. Again, the standard deviation of these values was taken as the resulting precision $\sigma _{{{{\mathrm{cluster}}}}}(N)$. Plotting *σ*_cluster_ as a function of *N* shows that, in the range of continuously decreasing FWHM—that is, for $1 < N < N_{{{\mathrm{c}}}}$—the median cluster spread rapidly scaled down to about 4 nm at *N* = *N*_*c*_ (Fig. 4a). For *N* > *N*_*c*_, *σ*_cluster_ improved more slowly because it scaled just with $1/\sqrt M$, with $M = N - N_{{{\mathrm{c}}}} + 1$ being the number of detections after the minimal FWHM had been reached. For *N* > 1,000, *σ*_cluster_ levels off at slightly below 1 nm, probably due to residual drift of the binding sites. Note that the measure *σ*_cluster_ includes, besides the pure MINSTED localization precision, all thermal and mechanical disturbances of both the microscope and the sample over the whole course of the experiment lasting for 40 minutes at room temperature.

Because a single localization took only ≈200 ms (Supplementary Fig. 9), estimating the localization precision on the basis of a single localization allowed us to reduce potential influences of movements. For each individual localization, the localization precision for $i \ge N_{{{\mathrm{c}}}}$ (at minimal radius *R*_min_) was obtained by calculating the standard deviation $\sigma _{{{{\mathrm{block}}}}}(M)$ of a moving mean of overlapping blocks of *M* center positions $\left( {x_i,y_i} \right)$. To ensure at least five independent data blocks, only blocks of size *M* < $(L - N_c + 1)/5$ were considered. To quantify single localization trace precisions up to the full number of detected photons, the values of $\sigma _{{{{\mathrm{block}}}}}(M)$ for $M = 1,\,2, \ldots ,(L - N_{{{\mathrm{c}}}} + 1)/5$ were fitted to a power law model $\sigma _{{{{\mathrm{est}}}}}\left( M \right) = a/\left( {b + M} \right)^c$ with parameters *a*,*b*,*c*. The parameter *b* > 0 accounts for correlations among short time spans caused by the fractional updates of the center positions, whereas $c \in \left( {0.4,\,0.5} \right]$ allows for the non-ideal use of photon information. To keep the two estimates $\sigma _{{{{\mathrm{block}}}}}\left( M \right)$ and $\sigma _{{{{\mathrm{est}}}}}(M)$ comparable within the whole range of photon numbers shown, only traces with $L - N_c + 1 \ge$ 10,000 are displayed, amounting to 39 traces in total. At 2,000 detected photons (including the ones with $R_i \ne R_{{{{\mathrm{min}}}}}$), a median localization precision of 4.7 Å was obtained from both estimators, which clearly indicates the ability of MINSTED to localize at a fraction of the fluorophore’s size of about 2 nm. The precision of 1 nm is attained with only 400 detections. With 10,000 detections in place and under the same realistic background and stability conditions, the estimated precision *σ*_est_ reached 2.3 Å (Fig. 4a).

Comparing the single localization estimate *σ*_est_ and the cluster analysis precision *σ*_cluster_ enabled us to assess the effective position uncertainty *s* of the binding sites over the 40 minutes of measurement as a proxy for the stability implicated in the process. By modeling $\sigma _{{{{\mathrm{cluster}}}}}\left( M \right) = \sqrt {\sigma _{{{{\mathrm{est}}}}}^2\left( M \right) + s^2}$, we obtained a value of *s* = 0.72 nm, which proves the long-term stability of our system. The combination of precision and stability enabled our blue-shifted MINSTED system to clearly resolve origami binding sites as close as 4 nm, which is about twice the molecular size of Cy3B (Fig. 4b–f). Registering all localizations with *L* > *N*_*c*_ resolved the entire origami pattern.

**Fig. 5:**
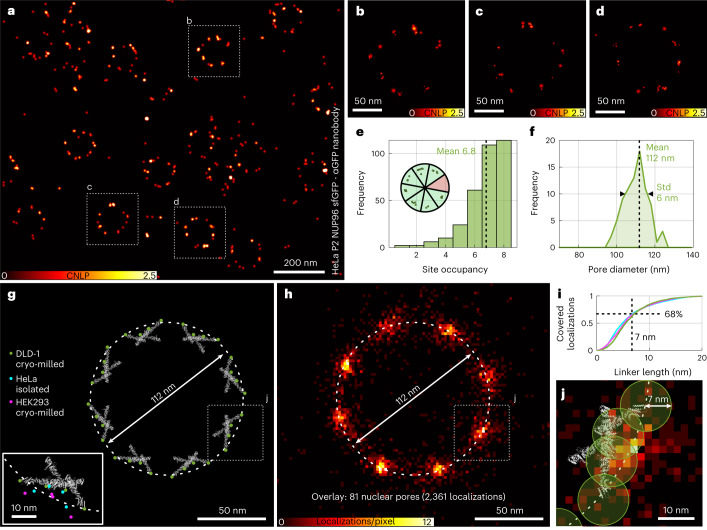
Blue-shifted MINSTED imaging of nuclear pores. **a**, MINSTED image of the nuclear surface of a HeLa P2 cell expressing nuclear pore protein NUP96 endogenously tagged with sfGFP and labeled with nanobody against GFP, offering a DNA binding site for hybridization with a complementary DNA strand labeled with the fluorophore Cy3B. **b**–**d**, Excerpts of individual NPC images from **a**, as indicated in the boxed region, highlighting the median localization precision of 0.9 nm. As NUP96 occurs in four copies per one-eighth of the eight-fold rotationally symmetric NPC, each ‘corner’ is expected to harbor up to four binding sites, which agrees well with the several individual dots in the images. **e**, Occupancy of the NPC’s eight asymmetric subunits displays a mean of 6.8. **f**, The average diameter formed by the Cy3B signal distribution is 112 ± 6 nm. **g**, NUP96 protein structure model extracted from cryo-ET data of NPCs from cryo-milled DLD-1 cells^11^. The 2D projected positions of the NUP96 C-termini are marked by dots in green (DLD-1), cyan (isolated HeLa NPCs^12^) and magenta (cryo-milled HEK293 NPCs^12^). **h**, Overlay image of 81 HeLa NPC MINSTED images renders the NPC’s expected eight-fold symmetry, with each corner displaying an elongation along the circumference, indicative of the slightly staggered arrangement of the NUP96 polypeptides adjacent to each other in each of the NPC’s octagonal subunits. **i**, Coverage of the obtained MINSTED localizations relative to the cryo-ET data, considering different distances between NUP96 and fluorophore (same colors as in **g**). **j**, Superimposition of the DLD-1 cryo-ET model and MINSTED overlay, considering a NUP96-to-fluorophore distance of 7 nm. **a**–**d** were rendered by displaying the individual localizations by Gaussian functions with an amplitude of unity and a standard deviation corresponding to the localization precision (Methods); we denote this as the cumulative normalized localization probability (CNLP).

To explore the performance of blue-shifted MINSTED nanoscopy in biological samples, we prepared mammalian (HeLa) cells expressing the nuclear pore protein NUP96 as polypeptides carboxy-terminally tagged with sfGFP serving as binding sites for anti-GFP nanobodies (Supplementary Fig. 6). Due to the ideally eight-fold symmetry of the nuclear pore complex (NPC) in the focal plane, NPC scaffold components like NUP96 are frequently used to assess the performance of nanoscopy methods^10^. The nanobodies targeting the GFP tags of these NUP96 polypeptides carried a DNA-docking strand to which a complementary DNA strand with a Cy3B fluorophore was able to bind by hybridization. About 30% of the localization attempts did not converge and were discarded (Supplementary Table 1 and Supplementary Fig. 4). The remaining 70% rendered images with an estimated median *σ* < 1 nm (Fig. 5a–d). Because the fluorescence images yield only the fluorophore distribution, they must be seen as a proxy of the actual NUP96 distribution in the cell. An estimated 3*σ* = 5–7 nm uncertainty is caused by the extent of the tag and the flexible peptide linking the tag to NUP96. This uncertainty should be contrasted with our 3*σ* < 3 nm fluorophore localization precision in cells, proving that, in MINSTED fluorescence nanoscopy, the main limits for extracting positional information of the biomolecules are set by the size and positional flexibility of the tags. Nonetheless, manually selecting all identifiable NPCs from the dataset (328 NPCs consisting of 8,116 localizations, which makes up 78% of all localizations from the dataset) and further analyzing their localization distribution yielded a mean site occupancy of 6.8 sites within the expected eight-fold arrangement (Fig. 5e). From this dataset, all NPCs with eight occupied sites were used for further analysis to ensure good coverage along the outline of the pore. Estimating their ellipticity, all NPCs with an aspect ratio >1.25 were excluded. From the remaining 81 NPCs, including 2,361 localizations (Supplementary Fig. 11), a mean diameter of 112 ± 6 nm was determined (Fig. 5f).

Next, we examined how our MINSTED images match with three cryo electron microscopy (cryo-EM) datasets supplying NUP96 structural information^11,12^. The *x*–*y*-projected structures of the 32 full-length NUP96 polypeptides per NPC are arranged according to their fitting into the cryo-EM map from cryo-milled DLD-1 NPCs. The NUP96 C-termini (amino acid 937) to which the tags are appended are highlighted (Fig. 5g). Evidently, the DLD-1 NUP96 C-termini are close to a circle with 112-nm diameter as anticipated. In addition, we extracted the C-termini positions of NUP96 polypeptides fitted into the cryo-EM maps of isolated HeLa and cryo-milled HEK293 NPCs. Comparing the datasets, differences in such positions are notable, hinting at potential variability of the proteins’ arrangements within NPCs depending on preparation, environment and cell type. Therefore, even without substantial linker offset, the question arises whether the fluorescence images of single NPCs each represent the averaged cryo-EM structure or also display physiologically relevant structural plasticity. From the 81 selected NPCs, an overlay image was created, which reproduced the NPC’s eight-fold symmetry as visualized via NUP96 (Fig. 5h). This overlay was used to estimate the fit of our data with the NUP96 positions deduced from the cryo-EM data. Because the GFP nanobody entity is appended to the NUP96 C-terminus via the flexible linker peptide, it can span, rotational freedom provided, a circular area of possible fluorophore positions in two dimensions (2D). At an estimated mean length of 7 nm, comprising linker, GFP and nanobody, a fraction of ≈68% of the localizations is included within those areas (Fig. 5i). Before this analysis, we excluded localizations with a radial distance of >20 nm from the mean diameter to compensate for perturbing localizations of binding sites from neighboring NPCs, unspecific binding of the nanobody or DNA strand (Supplementary Fig. 13). The elongated appearance of each NUP96 localization cluster along the periphery, as seen in the overlay image, can be interpreted as indicative of the slightly staggered arrangement of the NUP96 polypeptides within each of the NPC’s octagonal subunits^13^. Considering the possible fluorophore positions with a rotational radius of 7 nm, the cryo-EM structures (illustrated in Fig. 5j using DLD-1) and the data obtained by MINSTED are in good agreement (Fig. 5j and Supplementary Fig. 10).

Although labeling by DNA hybridization has many advantages over the use of photo-activation for on/off switching, blue-shifted MINSTED nanoscopy also works with the latter. This is demonstrated by labeling U-2 OS NUP96-Halo cells^10^ with the photo-activatable fluorophore Halo-ONB-CP560 (Supplementary Fig. 5a) having an absorption and emission maximum at 560 nm and 610 nm, respectively. Because each photo-activatable fluorophore provides just a single localization, the resulting NUP96 (Supplementary Fig. 5b) image displays a lower localization density. Here, a median *σ* of <2 nm was observed, whereas the median value of photons detected after reaching *R*_min_ was only 161. However, this example shows that, once photo-activatable fluorophores are optimized, precisions as with Cy3B can be attained throughout.

**Fig. 6:**
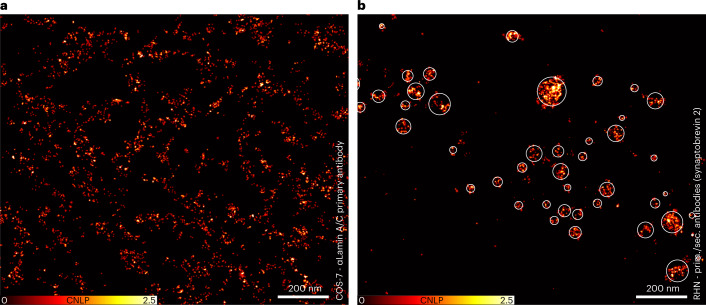
Blue-shifted MINSTED imaging of lamin and synaptic vesicles. **a**, Imaging of lamin A/C exemplifies the application of MINSTED at high labeling densities. **b**, MINSTED image of synaptic vesicles in neurons represented by primary and secondary antibody-tagged synaptobrevin 2. The shown clusters of single fluorophore events yield images of tagged synaptic vesicles that appear as entities of 37 ± 24 nm in diameter (4σ). Images were rendered by displaying the individual localizations by Gaussian functions with standard deviation corresponding to the localization precision (Methods). CNLP, cumulative normalized localization probability.

Having passed this test, MINSTED nanoscopy was next applied to structures not exhibiting a similarly symmetric arrangement of its components as the NPCs. For example, we visualized the nuclear lamina, the filamentous network that occurs positioned between the NPCs at the nuclear side of the nuclear envelope. Specifically, we labeled COS-7 cells with anti-lamin A/C antibodies having two DNA docking strands at their glycosylation sites close to the antibody binding pocket. The docking strands served for Cy3B labeling through DNA hybridization. The antibodies target the Ig-fold domain of lamin A/C. Recording the fluorophores and their positions for 68 minutes yielded a nanoscale proxy of the lamin distribution (Fig. 6a). The high binding site density infrequently caused undue binding of more than one fluorophore in the focal region, which we excluded (Supplementary Table 1 and Supplementary Fig. 4). Although this sample posed more challenges for separation and localization, the estimated median *σ* was <1 nm. Finally, we applied MINSTED to scrutinize the distribution of densely packed synaptic vesicles at the axon terminal in cultured rat hippocampal neurons by tagging the protein synaptobrevin 2 with a primary/secondary antibody sandwich. The resulting MINSTED image displays fluorophore clusters of 38 ± 23 nm in diameter, in line with the expectation to represent synaptic vesicles^14^ (Fig. 6b).

## Discussion

Summarizing our findings, blue-shifted MINSTED is able to localize individual fluorophores with *σ* = 1 nm precision using only 400 detected photons, a record low number. Note that, for this precision, an idealized background-free centroid-based localization would require ≈11,000 photons, underscoring the benefit of targeting the fluorophore with an intensity (donut) zero. Cutting down the number of required detections also reduces the influence of movements and drift. Fluorophores as close as 4 nm can be fully separated. Apart from discarding failed localizations, no further data post-processing or drift correction was required. This success is because (1) MINSTED localizes with a STED donut minimum providing a reference coordinate in the sample, which is (2) continuously moved closer to the fluorophore so that the de-excitation rate is kept constant; (3) STED and confocal detection suppress fluorescence background; and (4) low-power beams can be used to avoid subtle disturbances by heating.

As the 3*σ* value of 3 nm is even smaller than the size of the molecular construct linking the fluorophore to the biomolecule of interest, our results underscore that the limits of fluorescence nanoscopy applications in biology are no longer set by physical or technical factors but, rather, by the molecular tags and linkers. Evidently, a fluorescence image renders just the fluorophores, not the tagged biomolecules. This has to be considered when interpreting fluorescence microscopy data, especially when dealing with high localization precisions, as presented in this study. Moreover, the fluorophore positions are influenced not only by the linker length but also by the attachment site on the biomolecule itself and the steric constraints that the fluorophore might encounter at this position. Therefore, further advancements in molecule-scale biological imaging critically call for solutions to relate the position of the fluorophore to that of the target biomolecule. Finding such solutions has become more attractive than ever because MINSTED now enables localizations down to the Ångström domain. In fact, our analysis showed that if 10,000 emissions can be detected from the fluorophore under the same practical background and stability conditions, the precision is estimated to *σ* = 2.3 Å, a value that is about eight times smaller than the extent of the fluorophore itself. Clearly, once the problem of assigning the fluorophore’s position to that of the labeled target has been solved, such precisions should open up new pathways for studying biomolecular assemblies in cells with optical microscopes under physiological conditions.

Finally, we note that the attained precision records can still be improved without amending the MINSTED concept. As the detection rate is largely proportional to the pulse repetition rate of our diode lasers, increasing the rate from the present 10 MHz to 50–100 MHz should speed up the localization almost accordingly. As laser technology advances rapidly, this scenario may soon enable MINSTED localization with 1-nm precision in the millisecond domain, accommodating even faster movements and drifts. Likewise, fluorophore localizations with 1-Å precision should become routine.

## Methods

### MINSTED microscope

The core of the implemented MINSTED microscope is outlined in Supplementary Fig. 3, and its components are listed in the Supplementary Information. The microscope incorporates a laser beam scanning path and de-scanned confocal detection using two galvo scanners to address a field of 100 μm × 100 μm in the sample. The galvo scanners use an off-axis parabolic mirror relay^15^. This path contains a single-photon avalanche diode (APD2) for detecting fluorescence light emitted from the sample at 500–550-nm wavelength. Additionally, continuous-wave (cw) lasers with 473-nm and 561-nm wavelengths provide excitation light, whereas a 375-nm cw laser is used for the activation of caged fluorescent emitters. These three laser beams are s-polarized on the main polarizing beam splitter PBS1, which transmits the p-polarized laser beams from the second excitation path with ps-pulsed 560-nm excitation light and ns-pulsed 636-nm STED light. As there was no off-the-shelf pulsed STED laser available at 636-nm wavelength, we built the STED laser using commercial components: blue and red laser diodes, a pulsed diode laser driver and a Praseodymium-doped ZBLAN fiber for pulse amplification. The STED beam passes through a vortex phase plate to create the lateral STED doughnut. An achromatic quarter-wave retardation plate (λ/4) sets up the required circular polarization. The second excitation path features two electro-optical deflectors (EODx,y) to rapidly address a field of 2 μm × 2 μm without any mechanical movement. The control electronics and the driver of the EODs provide a bandwidth of 400 kHz, which is used to scan the beam in circles around the estimated position of the fluorophore at 125-kHz frequency. APD1 detects fluorescence light at 570–620-nm wavelength. The p-polarized fraction of the fluorescence light is fully de-scanned by the EODs. Its s-polarized fraction is partially de-scanned by the galvo scanner that samples the circles’ center positions but is too slow to follow the rapid circular scan trajectories. Ensuring equal path lengths of both the galvo path and the EOD path at APD1 enables the ability to gate the fluorescence signal.

Piezo stages move the sample over larger distances, such that the sample can be focused and the region of interest can be centered to the EODs’ image field. The sample position is actively stabilized by a dedicated three-axis piezo stage with sub-nanometer precision. The focus feedback signal is obtained by tracking the reflection of a 980-nm beam from the coverslip–sample interface on the z-lock camera CAM2. The lateral position feedback signal is obtained by tracking the images of fiducial markers on the x-y-lock camera CAM1. The fiducial markers are imaged in a field of about 40 μm × 40 μm off-axis to avoid interference with the imaged field of the sample. Both focus locks are polarization filtered to suppress stray light and reflections as much as possible. For the x-y-lock, a pupil filter with a central field block (FB) is used to block the direct reflection at the coverslip–sample interface. Infrared filters block the excitation and STED light below 850-nm wavelength.

The z-lock uses an 8-bit CMOS camera imaging the lateral position of the reflected beam at 800–1,500 frames per second (fps) depending on the extent of the selected region of interest. Sixteen consecutive camera images are binned and then processed to extract the beam center. The deviation of the beam center with respect to the target position is integrated and scaled to obtain the control signal. Including mechanical inertia, the closed-loop control bandwidth was 15–30 Hz.

The x-y-lock uses a 16-bit sCMOS camera imaging fiducials with 80–100 fps depending on the extent of the region of interest and exposure time. The fiducial positions are estimated by least squares fitting of their images to a 2D Gaussian profile with constant background^16^. The deviation of the lateral positions of trustworthy fiducials with respect to their initial positions is integrated and scaled to obtain the control signal. The closed-loop control bandwidth was about 40 Hz. Fiducials are considered trustworthy if neither their positions nor their intensities fluctuated noticeably.

For clarity, the polarization, spatial and spectral cleaning of the laser beams and their power modulations are simplified. The APDs, the lasers at 375-, 560-, 636- and 850-nm wavelength and the super-luminescent LED at 980-nm wavelength are pig-tailed or fiber-coupled to the system. The excitation laser at 561-nm wavelength is fed through a pinhole to cleanup the beam profile. All laser beams are linearly polarized. All beam powers can be modulated and/or shuttered internally or externally.

The microscope was controlled using a field-programmable gate array device and custom software implemented and executed with LabVIEW 2017 and MATLAB R2018b.

### Antibody conjugation

Primary antibody against lamin A/C (SAB4200236, Sigma-Aldrich / Merck) was modified with azides on the glycans by using the commercial GlyClick enzyme kit (L1-AZ1-025, Genovis). The azide-modified antibody (~200 µg in ~150 µl of Tris-buffered saline) was reacted with 50.4 nmol of DNA (5′-3′: TTA TAC ATC TA, Metabion, Planegg/Steinkirchen) bearing a dibenzocyclooctyne moiety on the 5′ end of the DNA for 48 hours and purified using a 10-kDa molecular weight cutoff filter (Vivaspin 500, Sartorius).

### Generation and characterization of a CRISPR–Cas9n-edited HeLa P2 cell line expressing NUP96-sfGFP

The human cervix adenocarcinoma HeLa sub-cell line P2 was described recently^17^. Tagging of the *NUP98-NUP96* alleles with the ORF for sfGFP^18^ was by the CRISPR–Cas9 double-nickase approach^19^, using one pair of single guide RNAs (sgRNAs) (*Hs*NUP96 sgRNA1, GTTGGGAGCCTGTGAGCCCC; *Hs*NUP96 sgRNA2, gCTCGCAGATAGGACTGGGTA) that were designed with a CRISPR Design tool^20^ provided online (http://www.e-crisp.org/E-CRISP/designcrispr.html). The sgRNAs without their protospacer adjacent motif (PAM) were cloned into the bicistronic Cas9n expression vector pSpCas9n(BB)-2A-Puro (PX462) V2.0 (ref. ^21^), kindly provided by Feng Zhang (Addgene plasmid no. 62987; http://n2t.net/addgene:62987), resulting in two sgRNA/Cas9n vectors for this integration site. All subsequent steps leading to the isolation of individual cell clones were performed as recently described for other genomically edited cell lines^22^. The subsequent characterization of the NUP96-sfGFP cell line by genomic PCR (*NUP96*-sequence-complementary, tag-flanking forward primer GTTGGTTCTGGCTGCATTTTTTACTTCC and reverse primer GGTCACAAGATCCAGAATGGCTAGGG), genomic sequencing, immunoblotting, live cell imaging and immunofluorescence microscopy was done as recently described^22^.

### Cell labeling

#### Sample 4 (sample numbers are specified in Supplementary Table 1)

Cells of line HeLa P2, endogenously expressing NUP96 carboxy-terminally tagged with sfGFP, were grown on coverslips in high-glucose DMEM (D6429, Sigma-Aldrich) with 10% (v/v) FBS (P40-37500; PAN-Biotech) and penicillin–streptomycin–amphotericin B solution (A5955, Sigma-Aldrich). After washes in warm PBS, the cells were fixed for 30 minutes with 2.4% of freshly prepared and methanol-free formaldehyde in PBS, followed by quenching with 50 mM NH_4_Cl in PBS for 5 minutes, subsequent permeabilization with 0.25% Triton X-100 in PBS for 5 minutes and blocking with 1% BSA in PBS for 30 minutes. Incubation with single-domain antibodies (art. no. 198, Massive-Taq-Q anti-GFP, Massive Photonics) against GFP, each with a single P3 DNA-PAINT docking site (5′-3′, TTT CTT CAT TA) coupled to it, was conducted in 1% BSA-containing PBS for 120 minutes. Unbound nanobodies were removed by three washes in PBS for 20 minutes each.

#### Sample 5

Human osteosarcoma cells of line U-2 OS, endogenously expressing NUP96 carboxy-terminally tagged with Halo-Tag^23^ (U-2 OS-CRISPR-NUP96-Halo clone no. 252, CLS GmbH) were grown in McCoy’s medium (16600082, Thermo Fisher Scientific) with 10% (v/v) FBS (S0615, Bio&SELL), 1% (v/v) sodium pyruvate (S8636, Sigma-Aldrich) and penicillin–streptomycin (P0781, Sigma-Aldrich) on coverslips. For fixation, the cells were treated with 8% (w/v) paraformaldehyde (PFA) in PBS at 37 °C for 5 minutes, quenched with 100 mM NH_4_Cl in PBS for 10 minutes and permeabilized using 0.5% (v/v) Triton X-100 in PBS for 5 minutes. Samples were incubated with Halo-ONB-CP560 (Supplementary Fig. 5) at 1 µM in PBS for 1 hour, followed by several washes with PBS for 1 hour. For sample alignment on the microscope, the samples were incubated with primary antibody against NUP153 (ab24700, Abcam), bearing Alexa Fluor 488 (A11001, Invitrogen / Thermo Fisher Scientific, 1:2,000 dilution) for 30 minutes in 2% (w/v) BSA in PBS. Finally, the cells were washed with PBS.

#### Sample 6

Monkey African green kidney COS-7 cells (Sigma-Aldrich, cat. 87021302, lot 05G008) were grown on coverslips in DMEM (31966047, Thermo Fisher Scientific) with 10% (v/v) FBS (S0615, Bio&SELL) and penicillin–streptomycin (P0781, Sigma-Aldrich). For fixation, the cells were treated with cold methanol (−20 °C) for 4 minutes. After blocking with 2% (w/v) BSA (A9418, Sigma-Aldrich) in PBS for 5 minutes, the cells were incubated with primary antibody conjugated with DNA in 2% (w/v) BSA in PBS for 1 hour and with secondary antibody bearing Alexa Fluor 488 (A11001, Invitrogen / Thermo Fisher Scientific, 1:2,000 dilution) for 30 minutes in the same buffer. Finally, the cells were washed with PBS. To ensure the specificity of the antibody used, Supplementary Fig. 7 shows a COS-7 cell nucleus prepared as described above but with an additional secondary antibody, carrying STAR RED and imaged with confocal and STED.

#### Sample 7

Cultured rat hippocampal neurons (obtained from Wistar rats) were prepared for MINSTED-DNA PAINT as described previously. Animal procedures were guided by the Max Planck Institute for Multidisciplinary Sciences Göttingen. Neurons (DIV 18) were fixed (4% PFA for 10 minutes), quenched for autofluorescence (100 mM NH_4_Cl_2_ for 10 minutes), permeabilized (5% Triton X-100 for 5 minutes) and blocked with serum proteins for 1 hour. Neurons were then treated with the primary antibody (synaptobrevin 2, 104008, SYSY, 1:250 dilution) overnight at 4 °C, followed by treatment with the secondary antibody coupled to a P3 DNA-PAINT strand (5′-3′, TTT CTT CAT TA, Massive Photonics, 1:100 dilution) for 1 hour at room temperature. After the antibody labeling, the samples were fixed again with 4% PFA for 5 minutes.

#### Sample 8

Human osteosarchoma cells U-2 OS (European Collection of Authenticated Cell Cultures, cat. 92022711, lot 17E015) were cultivated in McCoy’s medium (16600082, Thermo Fisher Scientific) with 10% (v/v) FBS (S0615, Bio&SELL), 1% (v/v) sodium pyruvate (S8636, Sigma-Aldrich) and penicillin–streptomycin (P0781, Sigma-Aldrich) on coverslips. For fixation, the cells were treated with cold methanol (−20 °C) for 4 minutes. After blocking with 2% (w/v) BSA (A9418, Sigma-Aldrich, 1:50 dilution) in PBS for 5 minutes, the cells were incubated with primary antibody against vimentin (V6389, Sigma-Aldrich) in 2% (w/v) BSA in PBS for 1 hour and with secondary antibody bearing Cy3B (515-005-003, Dianova) for 30 minutes in the same buffer. Finally, the cells were washed with PBS.

Before imaging, the cells were incubated with polyvinylpyrrolidone shelled silver nanoplates (SPPN980, nanoComposix) for 1 hour and washed with PBS.

### Single-molecule and DNA origami sample preparation

Polyvinnylpyrrolidone shelled silver nanoplates (SPPN980, nanoComposix) were diluted 1:500 in water, and 15 µl was dried on a coverslip that was previously cleaned with Hellmanex II (Hellma) and using a plasma cleaner operating with air. The coverslip was glued with double-sided scotch tape to a microscope slide to form a flow channel. This flow channel was rinsed with PBS and then filled with 15 µl of 0.5 mg ml^−1^ biotinylated BSA (A8549, Sigma-Aldrich) in PBS and incubated for 4 minutes before being washed with PBS. Subsequently, the channel was filled with 15 µl of 0.5 mg ml^−1^ streptavidin (11721666001, Sigma-Aldrich) in PBS and flushed after 4 minutes with PBS (10 mM MgCl_2_ in PBS for DNA origami samples). Details about this protocol are published in ref. ^2^.

For single-molecule samples, 15 µl of 200 pM double-stranded DNA consisting of one strand modified at the 5′ end with a biotin (5′-3′: TTA TTC CTC TAG TAT ATG GCA ATG AAA TTA T) and one strand bearing a Cy3B molecule at the 3′ end (5′-3′: TAA TTT CAT TGC CAT ATA CTA CAG GAA TAA) were pipetted into the channel and incubated for 4 minutes before the channel was washed with PBS. DNA origami samples were prepared in the same way using DNA origamis ordered from GATTAQuant diluted 1:2 in 10 mM MgCl_2_ in PBS.

### MINSTED imaging

The DNA-PAINT samples (samples 1–3 and 6) were mounted with variable amounts of Cy3B (2.5–15 nM; Supplementary Table 1) coupled to the 3′ end of the DNA oligonucleotide (P1 sequence: CTAGATGTAT, Metabion) in 200 µl of oxygen-deprived reducing-oxidizing buffer^24^. The buffer consisted of 100 µl reducing-oxidizing buffer (10% (w/v) glycose, 12.5% (v/v) glycerol, 0.1 mM TCEP, 1 mM ascorbic acid) and 100 µl of PBS supplemented with 2 µl of oxygen removal enzyme mix (25 units of pyranose oxidase (P4234, Sigma-Aldrich) and 80 µl of catalase (C100, Sigma-Aldrich) with 170 µl of PBS), 1 µl of 200 mM methyl viologen dichloride hydrate (856177, Sigma-Aldrich) and 75 mM magnesium chloride. Samples 4 and 7 were mounted with 2.5 nM and 5 nM of Cy3B coupled to the 3′ end of the DNA oligonucleotide (P3 sequence: GTAATGAAGA, Metabion) in PBS, respectively. Sample 5 was mounted in PBS.

For single-molecule imaging (E-PSF measurements), a confocal overview image was recorded, and isolated fluorophores were selected. Individual fluorophores were then centered and imaged in small fields (<250 nm × 250 nm) to minimize photo-bleaching when measuring the STED power-dependent E-PSF^25^.

For DNA origami imaging, bound strands were searched by confocal scanning for a field of interest until more than *N*_ON_ photons were detected in a 2 × 2 pixel neighborhood. To reliably differentiate immobilized strands from freely diffusing imager strands, a dwell time of 1.6 ms per pixel and a sample-specific threshold of $N_{{{{\mathrm{ON}}}}} \in \left[ {80,140} \right]$ were chosen depending on the sample background. When *N*_ON_ was exceeded, the MINSTED localization was initiated^3^. At a maximum pulse energy of *E*_max_ = 1 nJ, the FWHM was reduced to about 24 nm; thus, *R*_min_ was chosen at 12–15 nm accordingly. A localization was terminated if fewer than 16 detections were made within a time of 10–30 ms. As the STED beam minimizes the background during the localization, the termination count rate was set at about 5% of the initiation count rate. The filtering parameters, which were applied in advance of the further analysis, are displayed in Supplementary Table 1.

For cell imaging of nuclear pores, lamin A and synaptobrevin 2 (samples 4–7) by means of MINSTED DNA-PAINT, the search for binding sites was performed as mentioned above, whereas, for the localization, a maximum STED pulse energy of *E*_max_ = 0.5 nJ and a minimal scan radius of *R*_min_ = 15 nm (*R*_min_ = 20 nm for sample 5) was chosen. Localizations were aborted when fewer than 16 detections were made within 10–30 ms (as before) or latest after 200 ms (for samples 4, 6 and 7). This additional termination criterion was observed to reduce the DNA-PAINT docking site ‘bleaching’ (Supplementary Fig. 8). A possible explanation is that radical byproducts from fluorophore bleaching (which is reduced when limiting the maximum localization duration) might damage the docking sites. The filtering parameters are given in Supplementary Table 1. The distribution of fitting parameters among the localization events both before and after filtering is shown in Supplementary Fig. 4.

### Image analysis

The image analysis was performed using analysis tools implemented and executed with MATLAB R2020b and R2021b.

#### Rendering

The localizations shown in Figs. 4b,d,f, 5a–d and 6a,b and Supplementary Fig. 5b are each represented by a Gaussian centered at the estimated molecule position with a standard deviation according to the respective localization precision estimate. In Fig. 4b,d,f, the amplitude was set by normalizing each Gaussian to an area of unity. In Figs. 5a–d and 6a,b and Supplementary Fig. 5b, the amplitude of all localizations was set to 1. To ensure the visibility of highly precise localizations in large overview images, a minimum standard deviation for the displayed Gaussians was set to 3 nm, 1.5 nm and 1 nm in Figs. 5a and 6a,b, respectively. Those three subfigures, as well as Fig. 5b–d, were additionally saturated at a pixel value of 2.5 localizations for better visibility. The pixel size was set to 0.3 nm in Figs. 4b,d,f, 5a and 6a,b and to 0.1 nm in Fig. 5b–d and Supplementary Fig. 5b. Figures 4e and 5h,j are displayed as 2D histograms of estimated molecule positions with pixel sizes of 0.5 nm and 2 nm, respectively.

#### Particle overlay

Alignment of the 2D localizations of DNA origami images was performed using a formerly developed algorithm^26^. By means of translation and rotation, the algorithm searches for a best fit overlay of multiple realizations of the same structure (so-called particles) in a template-free manner. For Fig. 4e, all localizations with $L - N_c + 1 \ge 4,000$ were cut in as many as possible blocks of at least 2,000 elements (the first *N*_*c*_ detections were discarded). Subsequently, the filtering parameters from Supplementary Table 1 were applied. Clustering localizations around local maxima in a rendered and smoothed image with a maximum distance of 20 nm and a minimum number of 50 localizations resulted in 59 MINSTED images of isolated 3 × 3 origamis. After a first run of the particle alignment, resulting particles were rotated by manually chosen multiples of 60° to counteract the large periodicity in the 3 × 3 structure where outer binding sites were not yet well aligned. A second run of the particle alignment with the manually rotated particles as initial values resulted in the overlay as shown in Fig. 4e. For the nuclear pore statistics in Fig. 5, first all recognizable nuclear pores, even those that were very close to each other (see, for example, to the right from the center of Fig. 5a) were included. This resulted in a total number of 328 MINSTED images of nuclear pores with a total of 8,116 localizations, which were selected from a total region of ≈ 44 µm² (filtering was performed on the raw data as mentioned in Supplementary Table 1 for Fig. 5a–d). It is to mention that the full dataset consists of MINSTED measurements from multiple cell nuclei, which were consecutively recorded from the same sample. For the calculation of the site occupancy, the overlaid particles were each divided into eight zones (each zone covering a 45° sector of the circle). Every sector with at least one localization within a radial distance of 40–70 nm from the center was counted as occupied (Fig. 5e). To assure good angular coverage for the further analysis, we only considered pores with eight occupied sites. Additionally excluding pores with an ellipticity >1.25, we were left with a number of 81 pores including 2,361 localizations (Supplementary Fig. 11). For the calculation of the nuclear pore diameter, a circular ring with a radial Gaussian intensity profile was fitted to each of those selected pores (Fig. 5f). From the same dataset, the overlay in Fig. 5h was computed with the above-mentioned algorithm. As a prior result, the image in Supplementary Fig. 12 was obtained. In this, the eight-fold symmetry is well visible, whereas one of the clusters is strongly enhanced. This is caused by the objective function of the particle alignment algorithm, which prefers to map each particle’s site of highest density onto the global maximum of the overlay. To compensate for that, each of the 81 particles was rotated by a random multiple of 45°, and an additional particle alignment step that allowed for only small angular corrections was applied, which led to the final image as shown in Fig. 5h.

#### Cluster analysis

Cluster assignment of the data from Fig. 6b was performed by clustering regions of high localization density with an in-house-developed tessellation algorithm^27^. A detailed description is given in ref. ^14^. Nearby localizations were additionally assigned to their closest cluster if not farther away than 40 nm with respect to the cluster’s boundary. Clusters including fewer than ten localizations were discarded. The diameter of each cluster was estimated as four times the standard deviation of the positions of the assigned localizations $4\sqrt {\sigma _x\sigma _y}$.

#### Cryo-EM-based reconstruction models of the human NPC, for juxtaposition with MINSTED imaging

Three reconstruction models of the human NPC were used for comparing the current study’s MINSTED data with the C-termini positions of NUP96 in NPC structures determined by cryo electron tomography (cryo-ET). The one model is based on a cryo-ET map of cryo-milled NPCs of DLD-1 cells into which also experimentally determined protein structures had been fitted (Protein Data Bank entry 7PEQ (ref. ^11^)). The others are based on cryo-ET maps of NPCs of isolated HeLa cell nuclei and cryo-milled HEK293 cells (models kindly provided by Martin Beck) into which AI-predicted structures of NPC proteins had been modeled^12^, having used AlphaFold2 (refs. ^28,29^) and the ColabFold platform^30^ for the proteins’ structure predictions. To complete the NUP96 structure in the DLD-1 reconstruction model, into which a C-terminally truncated version of NUP96 had been fitted^11^, we aligned the AI-predicted full-length NUP96 structure, comprising aminos acids 1,207–1,817 of the human NUP98-NUP96 precursor protein (AF-P52948-F1-model_v2 in the AlphaFold database), with the DLD-1 model’s truncated NUP96 polypeptides, using the UCSF Chimera software package and its MatchMaker tool^31^. The obtained protein structure was rendered using UCSF ChimeraX (ref. ^32^).

### Statistics and reproducibility

The confocal and STED images in Fig. 2a,b were chosen from a total of seven images showing similar results, which were recorded from seven cells on one coverslip. The E-PSF in Fig. 2c resulted from the overlay of 478 frames taken from 44 single Cy3B molecules. The three remaining data points in Fig. 2d, measured with 0.1 nJ, 0.2 nJ and 0.5 nJ of STED pulse energy, resulted from 86, 105 and 215 frames from 25, 19 and 40 single Cy3B molecules, respectively. For Fig. 4a, a total number of 997 localizations from approximately 114 binding sites of 3 × 3 12-nm DNA origamis was recorded. Resulting from this dataset, 59 clusters were analyzed to obtain the blue data and 39 localizations to obtain the red data. This measurement was independently repeated at least three times, showing similar results. Figure 4b,c shows exemplary images of a single 3 × 3 6-nm DNA origami structure. Figure 4e resulted from the overlay of 59 3 × 3 6-nm DNA origami images. The image of one exemplarily chosen origami is shown in Fig. 4d. The image of the DNA origami structure, presented in Fig. 4f, was exemplarily chosen from a total number of 38 origami images. The NPC data, as shown in Fig. 5, resulted from a total of 11 nuclei on two coverslips. Figure 5a shows one exemplary image, and Fig. 5b–d shows zoom-ins to exemplarily chosen NPCs. For the overlay procedure (Fig. 5e,f,h,i,j), the whole dataset was taken into account (see above). The image in Fig. 6a was exemplarily chosen from a total of nine images from nine nuclei on three coverslips. The image in Fig. 6b was exemplarily chosen from a total of seven images from two coverslips.

### Reporting summary

Further information on research design is available in the Nature Research Reporting Summary linked to this article.

## Online content

Any methods, additional references, Nature Research reporting summaries, source data, extended data, supplementary information, acknowledgements, peer review information; details of author contributions and competing interests; and statements of data and code availability are available at 10.1038/s41587-022-01519-4.

## Supplementary information

Supplementary Information Calculation on large cross-section STED, MINSTED components list, EOD time-dispersion compensation and donor plasmid sequences; Supplementary References; Supplementary Table 1; and Supplementary Figs. 1–13

Reporting Summary

## Data Availability

The data that support the plots within this paper and other findings of this study were wrapped up and stored on an internal repository of the Max Planck Society. The data are available from the corresponding author (S.W.H.) upon reasonable request.
