# Supplementary material for: MINSTED nanoscopy enters the Ångström localization range
Source: Nat Biotechnol. 2022 Nov 7;41(4):569–76. doi: 10.1038/s41587-022-01519-4 (PMC10110459; doi:10.1038/s41587-022-01519-4)
Supplement: Supplementary file 1 — Calculation on large cross-section STED, MINSTED components list, EOD time-dispersion compensation and donor plasmid sequences; Supplementary References; Supplementary Table 1; and Supplementary Figs. 1–13 [file 41587_2022_1519_MOESM1_ESM.pdf]

nature biotechnology

Article https://doi.org/10.1038/s41587-022-01519-4

MINSTED nanoscopy enters the Ångström localization range

In the format provided by the authors and unedited

Supplementary Information

MINSTED nanoscopy enters the Ångström localization range

Michael Weber*[1], Henrik von der Emde*[1], Marcel Leutenegger[1], Philip Gunkel[2], Sivakumar Sambandan[3,4,1], Taukeer A. Khan[1], Jan Keller-Findeisen[1], Volker C. Cordes[2], Stefan W. Hell[1,5†]

[1]*Department of NanoBiophotonics, Max Planck Institute for Multidisciplinary Sciences, Am Fassberg 11, Göttingen 37077, Germany*
[2]*Department of Cellular Logistics, Max Planck Institute for Multidisciplinary Sciences, Am Fassberg 11, Göttingen 37077, Germany*
[3]*Synaptic Metal Ion Dynamics and Signaling, Max Planck Institute for Multidisciplinary Sciences, Am Fassberg 11, Göttingen 37077, Germany*
[4]*Laboratory of Neurobiology, Max Planck Institute for Multidisciplinary Sciences, Am Fassberg 11, Göttingen 37077, Germany*
[5]*Department of Optical Nanoscopy, Max Planck Institute for Medical Research, Jahnstraße 29, Heidelberg 69028, Germany*

**These authors contributed equally: Michael Weber and Henrik von der Emde*

† Correspondence: stefan.hell@mpinat.mpg.de

Large cross-section STED

Stimulated emission in STED is usually performed at the red tail of the emission spectrum of the respective fluorescent label to ensure that the STED light nearly exclusively turns fluorophores off (i.e. keeps them non-fluorescent), which is essential for separating fluorophores that are closer together than the diffraction limit. Choosing a STED wavelength closer to the emission maximum of the dye makes the stimulated emission cross-section $\varsigma_{\mathrm{OFF}}^{\mathrm{S}}$ larger, but reduces the contrast due to undesired excitation of the fluorescent dye by the STED light at the red tail of its absorption spectrum. Assuming a Boltzmann distribution of the occupation of the fluorescent molecules' vibrational levels and simplifying the excitation to target a single state only, the absorption cross-section $\varsigma_{\mathrm{ON}}^{\mathrm{S}}$ at the red tail of a fluorescent molecule with an absorption maximum λ_{abs} at temperature T follows

$$\varsigma_{\mathrm{ON}}^{\mathrm{S}}(\lambda) \propto \exp\left(-\frac{hc}{k_{\mathrm{B}}T}\left(\frac{1}{\lambda_{\mathrm{abs}}}-\frac{1}{\lambda}\right)\right), \tag{1}$$

where h, c and k_{B} represent Planck's constant, the speed of light and the Boltzmann constant, respectively. The absorption cross-section thus rapidly increases for wavelengths λ approaching λ_{abs} from the red tail. On the other hand, the stimulated emission cross-section scales as[1]

$$\varsigma_{\mathrm{OFF}}^{\mathrm{S}}(\lambda) \propto \lambda^4 E(\lambda), \tag{2}$$

where $E(\lambda)$ describes the emission (fluorescence) spectrum of the fluorophore. By tuning the STED wavelength, the effects of increased background due to direct excitation and enhanced STED efficiency can be balanced.

In order to obtain an estimate of the effective PSF shape under the influence of both stimulated emission and direct excitation by a donut shaped STED beam (denoted by superscript S) and a Gaussian shaped excitation beam (denoted by superscript E), we model the respective ON- and OFF- processes by the following rate equation

$$\frac{\partial}{\partial t} P_{\mathrm{ON}} = \left(k_{\mathrm{ON}}^{\mathrm{E}} + k_{\mathrm{ON}}^{\mathrm{S}}\right) P_{\mathrm{OFF}} - \left(k_{\mathrm{OFF}}^{\mathrm{S}} + k_{\mathrm{sp}}\right) P_{\mathrm{ON}} \,. \tag{3}$$

Herein, P_{ON} and P_{OFF} denote the space- and time-dependent probabilities of a fluorophore to occupy the ground (OFF) or the fluorescent (ON) state. k_{sp} is the spontaneous ON to OFF transition rate, while the induced rates are given by

$$k_{\text{final state}}^{\text{beam}} = \frac{I^{\text{beam}} \lambda^{\text{beam}} \varsigma_{\text{final state}}^{\text{beam}}}{hc} \,. \tag{4}$$

For time-invariant rates, equation (3) is easily solved. From an initial state $P_{\mathrm{ON}}(t = t_0) = P_0$, the evolution of the solution reads

$$P_{\mathrm{ON}}(t > t_0) = \frac{K_{\mathrm{ON}}}{K} + \left(P_0 - \frac{K_{\mathrm{ON}}}{K}\right) \mathrm{e}^{-K(t-t_0)} \,, \tag{5}$$

where $K_{\mathrm{ON}} = k_{\mathrm{ON}}^{\mathrm{E}} + k_{\mathrm{ON}}^{\mathrm{S}}$ denotes the accumulated ON rates and $K = k_{\mathrm{ON}}^{\mathrm{E}} + k_{\mathrm{ON}}^{\mathrm{S}} + k_{\mathrm{OFF}}^{\mathrm{S}} + k_{\mathrm{sp}}$ the sum of all rates.

From this, we now aim to analytically approximate the PSF shape created by a repetitive pulse sequence consisting of an excitation pulse followed by a STED pulse and finally a detection period in each cycle (Suppl. Fig. S1a). At time $t = 0$, the fluorophore shall be in its ground state ($P_{\mathrm{ON}}(0) = 0$). It shall then be exposed to an excitation pulse of duration τ^{E} and immediately thereafter to a STED pulse of duration τ^{S} (all pulse shapes assumed to be rectangular for the sake of simplicity). After the excitation pulse, the ON state probability is

$$P_{\mathrm{ON}}\left(\tau^{\mathrm{E}}\right) = \frac{k_{\mathrm{ON}}^{\mathrm{E}}}{k_{\mathrm{ON}}^{\mathrm{E}} + k_{\mathrm{sp}}} \left(1 - e^{-\left(k_{\mathrm{ON}}^{\mathrm{E}} + k_{\mathrm{sp}}\right)\tau^{\mathrm{E}}}\right) \approx k_{\mathrm{ON}}^{\mathrm{E}} \tau^{\mathrm{E}} \,, \tag{6}$$

where we assumed the excitation pulse to be short with respect to the fluorescence lifetime and the excitation to be weak, that is $\left(k_{\mathrm{ON}}^{\mathrm{E}} + k_{\mathrm{sp}}\right)\tau^{\mathrm{E}} \ll 1$. After the STED pulse, the ON state probability is

$$P_{\mathrm{ON}}\left(\tau^{\mathrm{E}} + \tau^{\mathrm{S}}\right) = \frac{K_{\mathrm{ON}}}{K} + \left(P_{\mathrm{ON}}\left(\tau^{\mathrm{E}}\right) - \frac{K_{\mathrm{ON}}}{K}\right) e^{-K\tau^{\mathrm{S}}} \propto I_{\mathrm{fl}} \,, \tag{7}$$

where I_{fl} approximates the time-gated fluorescence signal detected after the STED pulse, while $K_{\mathrm{ON}} = k_{\mathrm{ON}}^{\mathrm{S}}$ and $K = k_{\mathrm{ON}}^{\mathrm{S}} + k_{\mathrm{OFF}}^{\mathrm{S}} + k_{\mathrm{sp}}$ are the rates induced by the spontaneous decay and the STED pulse. We assume the laser pulse frequency to be much smaller than the spontaneous decay rate, i.e. all fluorophores will return to the ground state before the next cycle begins.

For estimating the peak SBR, we consider the fluorophore to be located (i) at the focal center, where the signal is induced only by the excitation beam, and (ii) at the STED donut crest, where the signal is mostly produced by the STED beam. The second position is defined at the maximum of the direct excitation signal produced by the STED beam (Suppl. Fig. S1b,d).

(i) At the focal center, $k_{\mathrm{OFF}}^{\mathrm{S}}$, $k_{\mathrm{ON}}^{\mathrm{S}}$ are negligible due to the absence of STED intensity in the donut center. Hence, the fluorescence signal reads

$$I_{\mathrm{fl}}^{\mathrm{center}} \propto k_{\mathrm{ON}}^{\mathrm{E}} \tau^{\mathrm{E}} \,. \tag{8}$$

(ii) At the donut crest, we assume strong depletion $k_{\mathrm{OFF}}^{\mathrm{S}} \tau^{\mathrm{S}} \gg 1$ of the ON state and the STED rates to be much larger than the spontaneous decay rate, i.e. $k_{\mathrm{OFF}}^{\mathrm{S}} + k_{\mathrm{ON}}^{\mathrm{S}} \gg k_{\mathrm{sp}}$. Therefore, we can simplify equation (7) by $K \approx k_{\mathrm{OFF}}^{\mathrm{S}} + k_{\mathrm{ON}}^{\mathrm{S}}$ and $e^{-K\tau^{\mathrm{S}}} \approx 0$, which yields the fluorescence signal

$$I_{\mathrm{fl}}^{\mathrm{crest}} \propto \frac{k_{\mathrm{ON}}^{\mathrm{S}}}{k_{\mathrm{OFF}}^{\mathrm{S}} + k_{\mathrm{ON}}^{\mathrm{S}}} = \varepsilon \,. \tag{9}$$

In an intermediate range, where the depletion of the excited state is weaker and the excitation stronger, we approximate equation (7) by

$$I_{\mathrm{fl}} \propto \varepsilon + \left(k_{\mathrm{ON}}^{\mathrm{E}}\tau^{\mathrm{E}} - \varepsilon\right)e^{-\left(k_{\mathrm{OFF}}^{\mathrm{S}}+k_{\mathrm{ON}}^{\mathrm{S}}+k_{\mathrm{sp}}\right)\tau^{\mathrm{S}}} \,. \tag{10}$$

The detection pinhole limits the signal collection to the central region within the donut crest. In this detection region, the induced fluorescence signal is composed of a constant pedestal ε plus a central peak, whose FWHM is determined by the exponential decay $e^{-K\tau^{\mathrm{S}}}$ and limited to the excitation FWHM d^{E} at weak depletion. This variable term is analogous to the typical confinement by STED. Assuming a Gaussian excitation profile and a quadratic profile near the center of the STED donut, an effective STED PSF with Gaussian profile is obtained for the center peak. Therefore, the center peak is confined to a FWHM[2]

$$d^{\mathrm{S}} = \frac{d^{\mathrm{E}}}{\sqrt{1 + \kappa K_{\mathrm{max}}\tau^{\mathrm{S}}}} \tag{11}$$

with $\kappa \approx 1$at d^{S} substantially smaller than d^{E}. $K_{\mathrm{max}}\tau^{\mathrm{S}}$ is the number of potential interactions during the STED pulse at the maximum STED intensity of the donut profile. From this, the full approximation of the effective excitation PSF at a distance r from the emitter position within the focal plane reads

$$I_{\mathrm{fl}}(r) \propto \varepsilon + \left(k_{\mathrm{ON}}^{\mathrm{E}}\tau^{\mathrm{E}} - \varepsilon\right)\exp\left(-4\ln(2)\,\left[\frac{r}{d^{\mathrm{S}}}\right]^2\right) \tag{12}$$

We attribute the constant pedestal ε to the background. The upper limit of the peak SBR for a single fluorescent emitter, estimated from (8) and (9) is therefore given by

$$\mathrm{SBR} < \frac{I_{\mathrm{fl}}^{\mathrm{center}}}{I_{\mathrm{fl}}^{\mathrm{crest}}} \approx \frac{k_{\mathrm{ON}}^{\mathrm{E}}\tau^{\mathrm{E}}}{\varepsilon}. \tag{13}$$

As indicated in Suppl. Fig. S1c, the approximations for the central excitation level, the direct excitation level, as well as the upper limit for the SBR are matching the theoretical description. In addition, the FWHM is decently approximated by equation (12) as shown in Suppl. Fig. S1d. For comparison, a simulated E-PSF is shown. The latter was numerically calculated by sequentially inserting the spatially dependent rates (diffraction patterns) in equation (5) while iterating through the steps of the pulse sequence. It thus seems reasonable to model the E-PSF as a Gaussian peak of FWHM d^{S} plus a constant base with an amplitude to yield a peak SBR as defined in equation (13). The drop of the E-PSF towards zero in the periphery can be modeled by the confocal pinhole, i.e. by multiplying the induced signal with the detection PSF.

In case of multiple emitters being active with an emitter density n but discernible in terms of the E-PSF's FWHM, another background term arises from these emitters being excited by the constant floor ε within the confocal detection area A.

$$\mathrm{SBR}(n) < \frac{I_{\mathrm{fl}}^{\mathrm{center}}}{I_{\mathrm{fl}}^{\mathrm{crest}} + \varepsilon n A} \approx \frac{k_{\mathrm{ON}}^{\mathrm{E}}\tau^{\mathrm{E}}}{\varepsilon(1 + nA)} \tag{14}$$

Suppl. Fig. S2 illustrates the issue that arises for STED imaging. Though imaging emitters of 30 nm distance with an E-PSF of 20 nm FWHM, resolution is drastically lost at an SBR of 10 for dense structures.

MINSTED microscope

Main components

375nm Diode laser iPulse-375-S-3V1 (18 mW cw), Toptica, Gräfelfing, Germany.

473nm Diode laser MBL-III-473-50 (50 mW cw), Changchun New Industries Optoelectronics, Changchun, China.

560nm	Pulsed fiber laser SuperK Extreme EXU-6 PP (600mW visible, 80 MHz, pulse picker), NKT Photonics, Köln, Germany.
561nm	Diode laser 85-YCA-010 (< 10 mW cw), Melles Griot, Rochester, NY, USA.
636nm	Diode laser HL63391DG (633-643nm, 200 mW), Ushio Opto Semiconductors, Tokyo, Japan; amplified in Praesodymium-doped fiber 201218/TB305 (Le Verre Fluoré, Bruz, France).
850nm	Super-luminescent LED module EBD273106-13 (< 5mW cw), Exalos, Schlieren, Switzerland.
980nm	Diode laser LuxX-980-150 (< 150 mW cw), Omicron-Laserage Laserprodukte, Rodgau-Dudenhofen, Germany.
$APD_{1,2}$	Single-photon counting module SPCM-NIR-3X-W4-FC, Excelitas, Wiesbaden, Germany.
CAM_1	USB3 sCMOS camera pco.panda 4.2 (2048×2048 pixels 6.5×6.5 μm^2, 16 bits, 100 fps), PCO, Kelheim, Germany.
CAM_2	USB3 CMOS camera xiQ MQ013RG-ON (1280×1024 pixels 4.8×4.8 μm^2, 8/10 bits, 210 fps), Ximea, Münster, Germany.
$EOD_{x,y}$	Electro-optic deflectors 311A (AD*P, Ø2 mm, 200 mm long, 7 µrad/V, 180 pF, ±500 V), Conoptics, Danbury, CT, USA; high-voltage drivers PZD700A-1-H-SHV-CE (±700 V, 200 mA, 150 kHz), Trek, Lockport, NY, USA.
FPGA	PCIe board 7852R with drivers and software LabVIEW 2017, National Instruments, Austin, TX, USA.
$G_{1,2}$	Galvo-mirrors 6 mm × 10 mm, galvo 6215H, servo drivers 671, Cambridge Technology, Bedford, MA, USA.
Objective	Magnification HCPL APO CS2 100×/1.4 NA oil-immersion, Leica Mikrosysteme, Wetzlar, Germany.
Stages	Three-axis piezo stage P-562.3CD with controller E-727.3CD on two-axis piezo stage M-686.D64 with controller C-867.260 and single-axis piezo stage M-230.25 with controller C-863.11, Physik Instrumente, Karlsruhe, Germany.

Filters

FB	Field block R1DF100, Thorlabs, Newton, NJ, USA.
PH_1	Pinhole Ø50 µm, Linos Photonics, Göttingen, Germany.
PH_2	Pinhole Ø100 µm, Linos Photonics, Göttingen, Germany.
VPP	Vortex phase plate V-660-20-1, Vortex Photonics, München, Germany.
F_1	VersaChrome edge tuneable long-pass filter TLP01-628, Semrock, Rochester, NY, USA.
F_2	Excitation filter ZET561/10x, Chroma Technology, Bellows Falls, VT, USA.
$F_{3,7}$	BrightLine single-band band-pass filter FF01-582/75, Semrock, Rochester, NY, USA.
F_4	VersaChrome edge tuneable short-pass filter TSP01-628, Semrock, Rochester, NY, USA.
F_5	Razor-edge ultrasteep long-pass filter LP02-561RE, Semrock, Rochester, NY, USA.
F_6	Razor-edge ultrasteep short-pass filter SP01-633RU, Semrock, Rochester, NY, USA.
F_7	BrightLine multiphoton short-pass filter FF01-680, Semrock, Rochester, NY, USA.
F_8	EdgeBasic long-pass filter BLP01-488R, Semrock, Rochester, NY, USA.
F_9	Emission band-pass filter HQ525/50, Chroma Technology, Bellows Falls, VT, USA.

Lenses

C_1	Fibre collimator 15 mm, 60FC-A15-4-01, Schäfter+Kirchhoff, Hamburg, Germany.
C_2	Fibre collimator 18 mm, 60FC-A18-0-01, Schäfter+Kirchhoff, Hamburg, Germany.
C_3	Achromatic doublet 15 mm, 84-328, Edmund Optics, Mainz, Germany.
C_4	Fibre collimator 7.5 mm, 60FC-A7.5-0-01, Schäfter+Kirchhoff, Hamburg, Germany.
C_5	Fibre collimator 4.5 mm, 60FC-A4.5-0-01, Schäfter+Kirchhoff, Hamburg, Germany.
C_6	Achromatic doublet 20 mm, NIR, AC080-020-B, Thorlabs, Newton, NJ, USA.
C_7	Achromatic doublet 10 mm, 322206000, Linos Photonics, Göttingen, Germany.
$L_{1,9}$	Achromatic doublets 400 mm, 322275322, Linos Photonics, Göttingen, Germany.
L_2	Achromatic doublet 80 mm, 322323000, Edmund Optics, Mainz, Germany.

$L_{3,5,15,16}$ Achromatic doublets 100 mm, 322345000, Linos Photonics, Göttingen, Germany.
L_4 Achromatic doublet 100 mm, 49-333, Edmund Optics, Mainz, Germany.
L_6 Achromatic doublet 300 mm, 322273322, Linos Photonics, Göttingen, Germany.
L_7 Achromatic doublet 150 mm, 322331322, Linos Photonics, Göttingen, Germany.
L_8 Achromatic doublet 50 mm, 322339000, Linos Photonics, Göttingen, Germany.
$L_{10.1,10.2}$ Achromatic doublets 150 mm, AC254-150-B, Thorlabs, Newton, NJ, USA.
$L_{11,12}$ Achromatic doublets 500 mm, 322329000, Linos Photonics, Göttingen, Germany.
$L_{13,14}$ Achromatic doublets 100 mm, 322236000, Linos Photonics, Göttingen, Germany.
L_{17} Achromatic doublet 300 mm, AC254-300-B, Thorlabs, Newton, NJ, USA.
$L_{18,19}$ Achromatic doublets 50 mm, 322265525, Linos Photonics, Göttingen, Germany.

Mirrors

CM Cold mirror M254C45, Thorlabs, Newton, NJ, USA.
DM_1 Dichroic mirror Z670SPRDC, Chroma Technology, Bellows Falls, VT, USA.
$DM_{2,4}$ Dichroic mirror FF560-fDi02-t3, Semrock, Rochester, NY, USA.
DM_3 Dichroic mirror H405LPXR, Chroma Technology, Bellows Falls, VT, USA.
DM_5 Dichroic mirror 495DCXR, Chroma Technology, Bellows Falls, VT, USA.
DM_6 Dichroic mirror 555DCXRU, Chroma Technology, Bellows Falls, VT, USA.
$PM_{1,2}$ Off-axis parabolic mirror MPD129-P01, Thorlabs, Newton, NJ, USA.
Unmarked MaxMirror ultra-broadband mirror, Semrock, Rochester, NY, USA.

Polarization optics

$PBS_{1,2}$ Polarizing beam splitter cube 49-002, Edmund Optics, Mainz, Germany.
PBS_3 Polarizing beam splitter cube PBS202, Thorlabs, Newton, NJ, USA.
PBS_4 Polarizing beam splitter cube PTW 2.20, Bernhard Halle, Berlin, Germany.
λ/4 Achromatic quarter-wave retarder plate RAC 3.4.10, Bernhard Halle, Berlin, Germany.
λ/2 Achromatic half-wave retarder plates RAC 3.2.10, Bernhard Halle, Berlin, Germany.

Compensating time-dispersion of electro-optical deflector response

Besides the near-instantaneous response, electro-optic materials exhibit creep due to relaxation processes in the material, which results in a gradual settling of the response when a voltage is applied for tens of milliseconds. We implemented a bi-exponential filter to compensate this behavior by the applied voltages. As a result, the responses of our AD*P deflectors were linearized for low frequencies up to several kHz and showed a residual settling $< 10^{-3}$ of the commanded deflection. A detailed description of both methods and results was carried out[3].

Donor plasmid sequence

NUP96 exon 33 homology arms for creating the donor plasmid with an insertion site (AgeI/BsrGI) for the ORF of the sfGFP-tag (gene synthesis product):

CGGGATCTCAAGACTTTTCCTAGTCCAAGGCTAGGTAACTGTGTTACCTTCCTCTTGGCTATTACTGCATAATTAGTGCCTTGTC
CTCCACTAGATGGTGGTGGCTTGGCCCTGTGTCATCATCTTGGATTTTCCCCTCCCTCACCTCACTGTTGTTTCAAGGTTTTGTG
TAGAGTCTATAGGTGGGATTGGAGTGATAGGAACTCCCCTTGGATTAATTGGCTTCTCTGCTTCTTTGTAGGTGGATTGCTCA
GGTAATGACCTGGAGCAGTTACACATCAAAGTGACTTCACTGTGCAGTCGGATAGAGCAGATTCAGTGTTACAGTGCTAAAG
ATCGCCTGGCTCAGTCAGGTAAGCCTCTAACCTCCTCACTCTTTCTGCCTTCTTGCTTCCTGTTTTTATGATTATTACACCCCACC
CTCAGTGCCTACCACCCTTCTCCAGACCCCATGCTCAGTGCTTGACTCTAGTTTTTCTCTCTAGACATGGCCAAACGTGTAGCCA
ACCTGCTGCGCGTGGTGCTGAGTCTGCATCATCCTCCTGATAGAACCTCCGACTCAACACCAGACCCTCAGCGAGTCCCTTTG
CGCCTCTTGGCTCCCCACATTGGCCGGCTTCCCATGCCTGAGGACTATGCCATGGACGAACTGCGCAGCCTTACCCAGTCCTA
TCTGCGAGAACTGGCTGTTGGGAGCCTGGGATCTGCTGGCTCCGCTGCTGGTTCTGGCACCGGTTCGGCATGGACGAACTGT
ACAAGTGAGCCCCAGGCACTTTGCATCACAGTCACATGCCCATTCACACCACACAGAGGTTCCCTGCCTTGTTTGGATTGGCA
CTGTTTGCCATTCTCTGGGTTGGCTGTGGCATCTACCCTCCCTCCCTGCTGCCAGAAGCAGCATCCTCCACTTGTTCAGGGCTT
TTCTTAATACTGAACGTAGCATAAGGGCTTCTGGAACCCAGAAGAGGAGACAGTTTACCATCCTCAAGATCATTCAGTGTTTT
TCCTTTAAAAAAATGGTCAATAAAGCTCCTTTGGCAGAATCCCCCAAAGAACCAGGGTATTCTTTTTCCATCCCTAGCCATTCT
GGATCTTGTGACCCTCCATGCCAACCAGCTTCCCTACTCCTACCCTGGCCCTTTTATACTAGGACTCCTTAGGAGGAGTGAGAC
AGGTGATAATGGATCCTTAACAGATGAACTATCCACAGAAGGAAGAGGGATCCGTCTCTTAAGTAATTGGTTAGTTAACACT
GAATTTTGGAGGCAAAGGAGGGTTGGCCTGAGTTAGGAAACAAAATGGGATCTTTCTGACACACTTAGGGCAGAAGTGAAT
GCCTGTCACGGAGGGATTGATCTTCAGGGCTGTTTTTGTTCCTGCCTTTAGAGTTCCATGAACACCATACCTTTGCTACTACTA
TGTGCAGGAACCCTTGGTCACATGTGACATGTCTGTGGG

Final sequence with inserted ORF for sfGFP-tag:

CGGGATCTCAAGACTTTTCCTAGTCCAAGGCTAGGTAACTGTGTTACCTTCCTCTTGGCTATTACTGCATAATTAGTGCCTTGTC
CTCCACTAGATGGTGGTGGCTTGGCCCTGTGTCATCATCTTGGATTTTCCCCTCCCTCACCTCACTGTTGTTTCAAGGTTTTGTG
TAGAGTCTATAGGTGGGATTGGAGTGATAGGAACTCCCCTTGGATTAATTGGCTTCTCTGCTTCTTTGTAGGTGGATTGCTCA
GGTAATGACCTGGAGCAGTTACACATCAAAGTGACTTCACTGTGCAGTCGGATAGAGCAGATTCAGTGTTACAGTGCTAAAG
ATCGCCTGGCTCAGTCAGGTAAGCCTCTAACCTCCTCACTCTTTCTGCCTTCTTGCTTCCTGTTTTTATGATTATTACACCCCACC
CTCAGTGCCTACCACCCTTCTCCAGACCCCATGCTCAGTGCTTGACTCTAGTTTTTCTCTCTAGACATGGCCAAACGTGTAGCCA
ACCTGCTGCGCGTGGTGCTGAGTCTGCATCATCCTCCTGATAGAACCTCCGACTCAACACCAGACCCTCAGCGAGTCCCTTTG
CGCCTCTTGGCTCCCCACATTGGCCGGCTTCCCATGCCTGAGGACTATGCCATGGACGAACTGCGCAGCCTTACCCAGTCCTA
TCTGCGAGAACTGGCTGTTGGGAGCCTGGGATCTGCTGGCTCCGCTGCTGGTTCTGGCACCGGTTCCATGGTGAGCAAGGGC
GAGGAGCTGTTCACCGGGGTGGTGCCCATCCTGGTCGAGCTGGACGGCGACGTAAACGGCCACAAGTTCAGCGTGCGCGGC
GAGGGCGAGGGCGATGCCACCAACGGCAAGCTGACCCTGAAGTTCATCTGCACCACCGGCAAGCTGCCCGTGCCCTGGCCC
ACCCTCGTGACCACCCTGACCTACGGCGTGCAGTGCTTCAGCCGCTACCCCGACCACATGAAGCGCCACGACTTCTTCAAGTC
CGCCATGCCCGAAGGCTACGTCCAGGAGCGCACCATCAGCTTCAAGGACGACGGCACCTACAAGACCCGCGCCGAGGTGAA
GTTCGAGGGCGACACCCTGGTGAACCGCATCGAGCTGAAGGGCATCGACTTCAAGGAGGACGGCAACATCCTGGGGCACAA
GCTGGAGTACAACTTCAACAGCCACAACGTCTATATCACCGCCGACAAGCAGAAGAACGGCATCAAGGCCAACTTCAAGATC
CGCCACAACGTGGAGGACGGCAGCGTGCAGCTCGCCGACCACTACCAGCAGAACACCCCCATCGGCGACGGCCCCGTGCTG
CTGCCCGACAACCACTACCTGAGCACCCAGTCCGTGCTGAGCAAAGACCCCAACGAGAAGCGCGATCACATGGTCCTGCTGG
AGTTCGTGACCGCCGCCGGGATCACTCACGGCATGGACGAGCTGTACAAGTGAGCCCCAGGCACTTTGCATCACAGTCACAT
GCCCATTCACACCACACAGAGGTTCCCTGCCTTGTTTGGATTGGCACTGTTTGCCATTCTCTGGGTTGGCTGTGGCATCTACCC
TCCCTCCCTGCTGCCAGAAGCAGCATCCTCCACTTGTTCAGGGCTTTTCTTAATACTGAACGTAGCATAAGGGCTTCTGGAACC
CAGAAGAGGAGACAGTTTACCATCCTCAAGATCATTCAGTGTTTTTCCTTTAAAAAAATGGTCAATAAAGCTCCTTTGGCAGA
ATCCCCCAAAGAACCAGGGTATTCTTTTTCCATCCCTAGCCATTCTGGATCTTGTGACCCTCCATGCCAACCAGCTTCCCTACTC

CTACCCTGGCCCTTTTATACTAGGACTCCTTAGGAGGAGTGAGACAGGTGATAATGGATCCTTAACAGATGAACTATCCACAG
AAGGAAGAGGGATCCGTCTCTTAAGTAATTGGTTAGTTAACACTGAATTTTGGAGGCAAAGGAGGGTTGGCCTGAGTTAGG
AAACAAAATGGGATCTTTCTGACACACTTAGGGCAGAAGTGAATGCCTGTCACGGAGGGATTGATCTTCAGGGCTGTTTTTG
TTCCTGCCTTTAGAGTTCCATGAACACCATACCTTTGCTACTACTATGTGCAGGAACCCTTGGTCACATGTGACATGTCTGTGG
G

Supplementary references

1. Beaumont, P.C., Johnson, D.G. & Parsons, B.J. Photophysical properties of laser dyes: picosecond laser flash photolysis studies of Rhodamine 6G, Rhodamine B and Rhodamine 101. *Journal of the Chemical Society, Faraday Transactions* **89**, 4185-4191 (1993).
2. Leutenegger, M., Eggeling, C. & Hell, S.W. Analytical description of STED microscopy performance. *Optics express* **18**, 26417-26429 (2010).
3. Leutenegger, M., Weber, M., von der Emde, H. & Hell, S.W. Compensating the delayed electro-optical deflector response. *arXiv preprint arXiv:2112.01621* (2021).
4. Fontoura, B.M., Blobel, G. & Matunis, M.J. A conserved biogenesis pathway for nucleoporins: proteolytic processing of a 186-kilodalton precursor generates Nup98 and the novel nucleoporin, Nup96. *The Journal of cell biology* **144**, 1097-1112 (1999).
5. Landry, J.J. et al. The genomic and transcriptomic landscape of a HeLa cell line. *G3: Genes, Genomes, Genetics* **3**, 1213-1224 (2013).
6. Adey, A. et al. The haplotype-resolved genome and epigenome of the aneuploid HeLa cancer cell line. *Nature* **500**, 207-211 (2013).
7. Hase, M.E. & Cordes, V.C. Direct Interaction with Nup153 Mediates Binding of Tpr to the Periphery of the Nuclear Pore Complex. *Molecular Biology of the Cell* **14**, 1923-1940 (2003).
8. Cordes, V.C., Reidenbach, S., Rackwitz, H.-R. & Franke, W.W. Identification of Protein p270/Tpr as a Constitutive Component of the Nuclear Pore Complex–attached Intranuclear Filaments. *Journal of Cell Biology* **136**, 515-529 (1997).
9. Gunkel, P., Iino, H., Krull, S. & Cordes, V.C. ZC3HC1 Is a Novel Inherent Component of the Nuclear Basket, Resident in a State of Reciprocal Dependence with TPR. *Cells* **10**, 1937 (2021).
10. Krull, S. et al. Protein Tpr is required for establishing nuclear pore-associated zones of heterochromatin exclusion. *The EMBO Journal* **29**, 1659-1673 (2010).

Supplementary figures and tables

Suppl. Tab. T1 | Sample preparation and filtering parameters.

Figure	Sample	Sample No.	Imager conc. [nM]	Filtering parameters: Minimal N	Maximal σ_x, σ_y [nm]	Minimal count rate [cps]	Maximal count rate [cps]	Events discarded
4a blue	3×3 12 nm grid	1	7.5	-	4.2	-	-	102 (10 %)
4a red				10’000	4.2	-	-	958 (96 %)
4b,c				-	-	-	-	0 (0 %)
4d	3×3 6 nm grid	2	15	-	-	-	-	0 (0 %)
4f	5×1 4/10 nm rod	3						
4e	3×3 6 nm grid	2	15	100	5.5	-	16’000	1’588 (19 %)
5a-d,h,j	HeLa P2 Nup96 sfGFP	4	2.5	100	6.5	5’000	16’500	291 (23 %)
S5b	U-2 OS Nup96 Halo	5	-	10	8	3’000	25’000	292 (45 %)
6a	COS-7	6	5	10	7	8’000	18’500	5’117 (30 %)
6b	Cultured rat hippocampal neurons	7	5	100	10	4’000	22’000	993 (21 %)
2a,b	U-2 OS	8	-	-	-	-	-	-

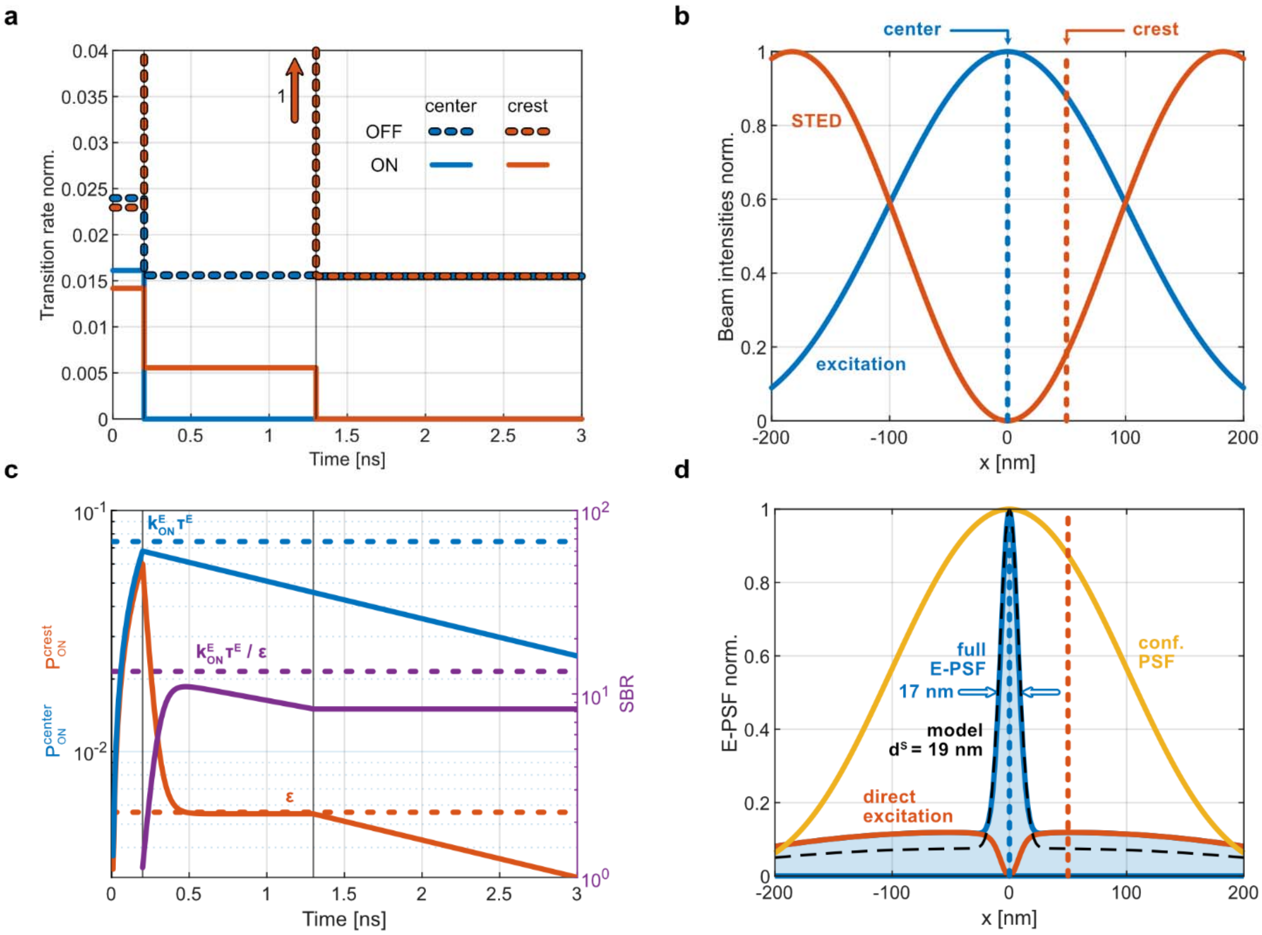

Suppl. Fig. S1 | Simulation of short-wavelength STED E-PSF. a The time-dependent accumulated ON- and OFF-rates are plotted for an excitation of Cy3B molecules at 560 nm wavelength with 1.5 µW of power for a pulse duration of 200 ps at 20 MHz repetition rate and a subsequently following STED pulse of 610 nm wavelength at 18 mW of power. **b** The diffraction patterns of the excitation and STED beams are plotted and the center and crest positions are displayed. The crest was defined by the maximum of the 'direct' excitation pedestal. **c** ON-state occupation probability at the center and at the crest are shown throughout the pulse sequence and the resulting SBR is plotted. For comparison, the approximations given in the text are compared to the theoretical model. **d** The confocal PSF, the E-PSF, as well as the 'direct' excitation pedestal and the remaining center peak are shown. For comparison, the approximation model is shown, consisting of a Gaussian peak of FWHM d^S with a constant pedestal in order to match the approximated SBR and multiplied by the detection PSF.

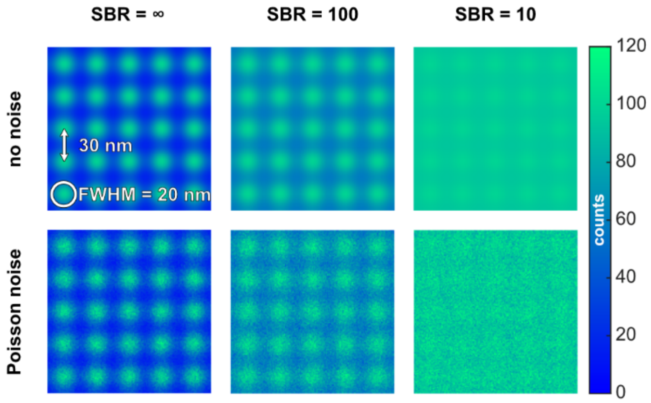

Suppl. Fig. S2 | Simulated STED imaging of fluorophores arranged in a grid with 30 nm spacing using E-PSFs with a peak FWHM of 20 nm. The different 'direct' excitation pedestals were set by the indicated SBRs. The E-PSFs were modeled with the 'direct' excitation pedestal $\exp(-k\boldsymbol{r}^2)\,/(1+2\exp(-k\boldsymbol{r}^2))$ added to a central Gaussian peak. The E-PSFs matched simulated PSFs very well if *k* was chosen to make up a Gaussian of FWHM d^{E}.

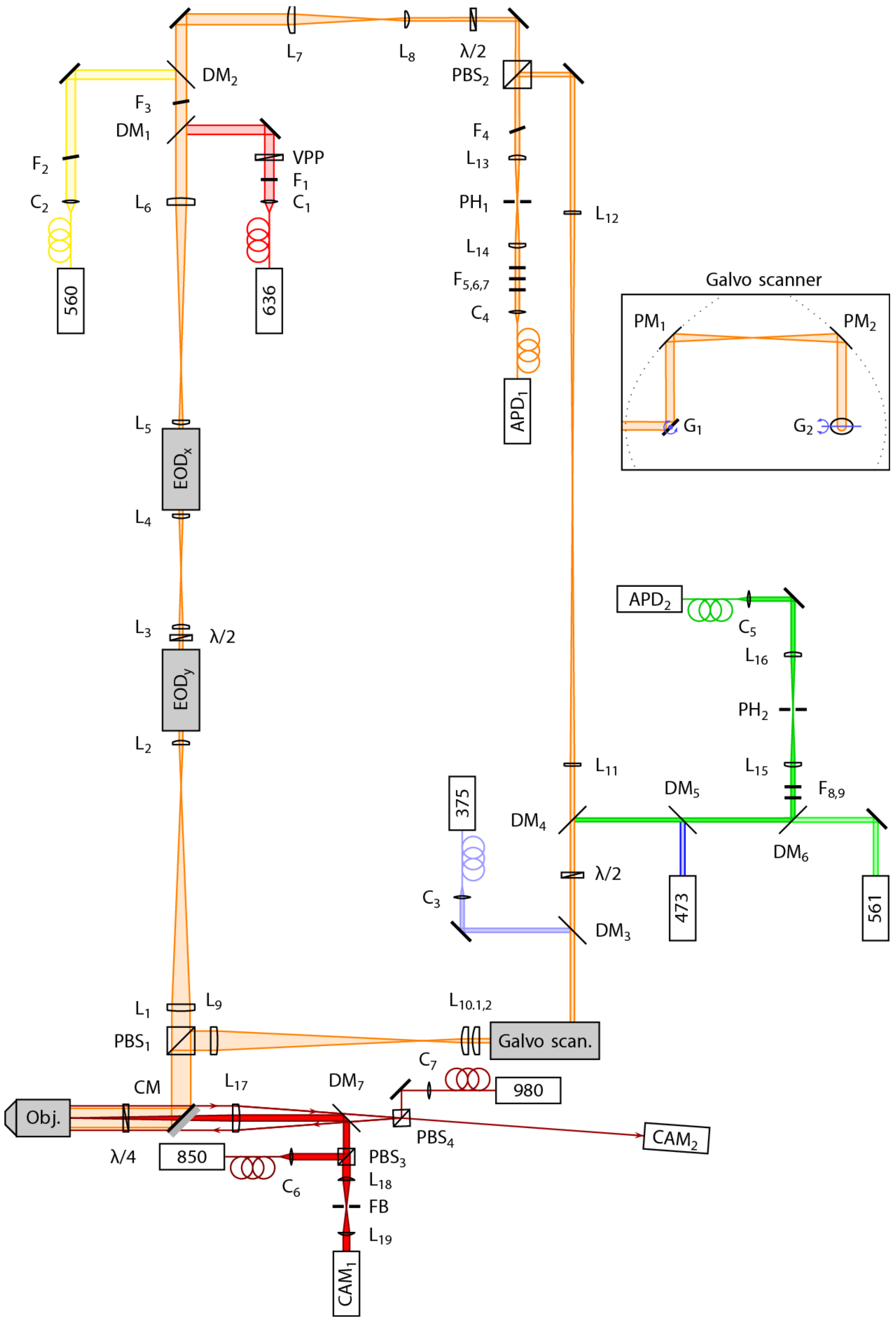

Suppl. Fig. S3 | Simplified scheme of the MINSTED microscope.

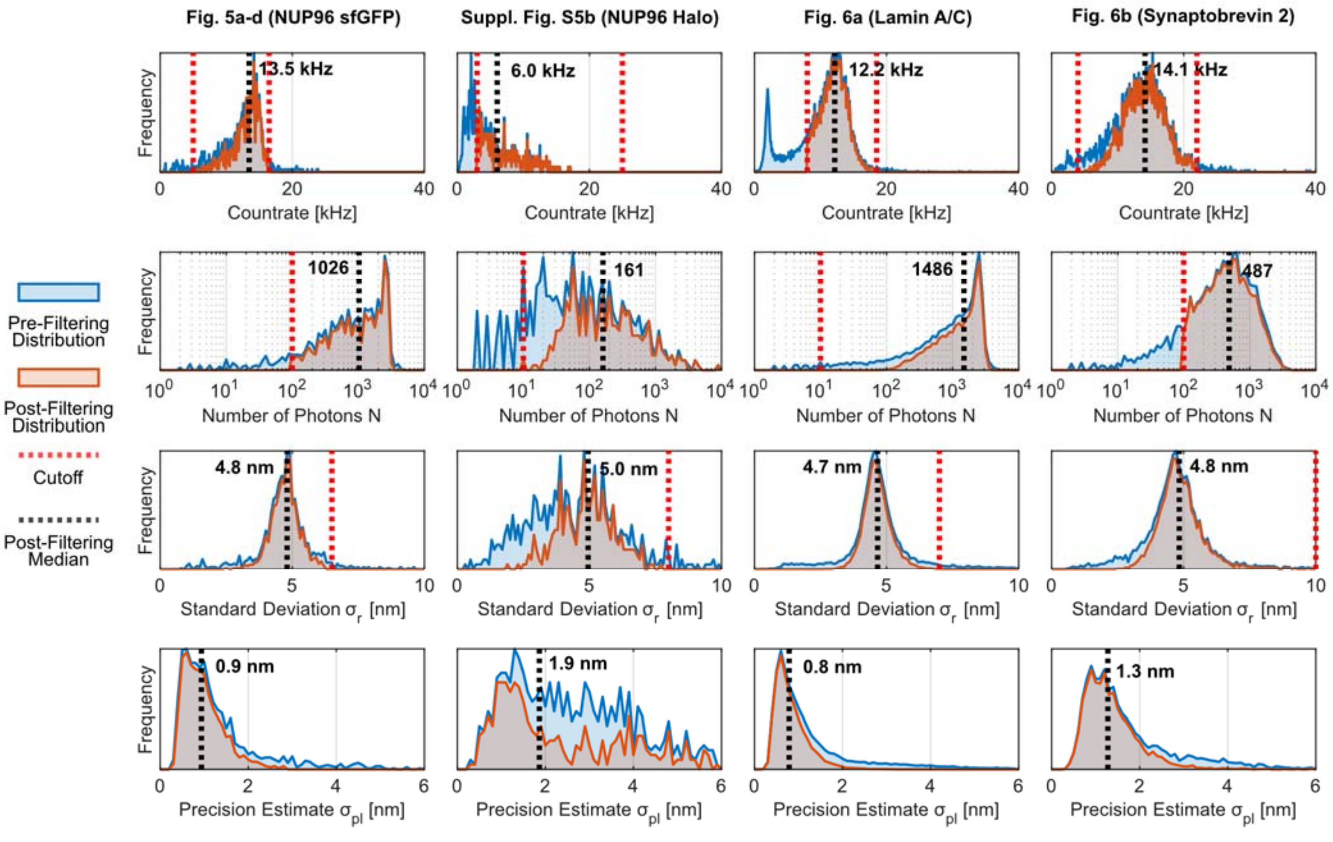

Suppl. Fig. S4 | Distribution of filtering parameters and estimated localization precisions before and after filtering. The data is shown for those datasets displayed in Fig. 5 and Fig. 6. Among the recorded localizations (with at least N_c detections) the distribution of the countrate (photons per time), the number of photons at minimal circling radius, the standard deviation of center positions at minimal circling radius (the geometric mean of the x- and y-coordinate standard deviations is shown) and the precision estimate σ_{est} are presented.

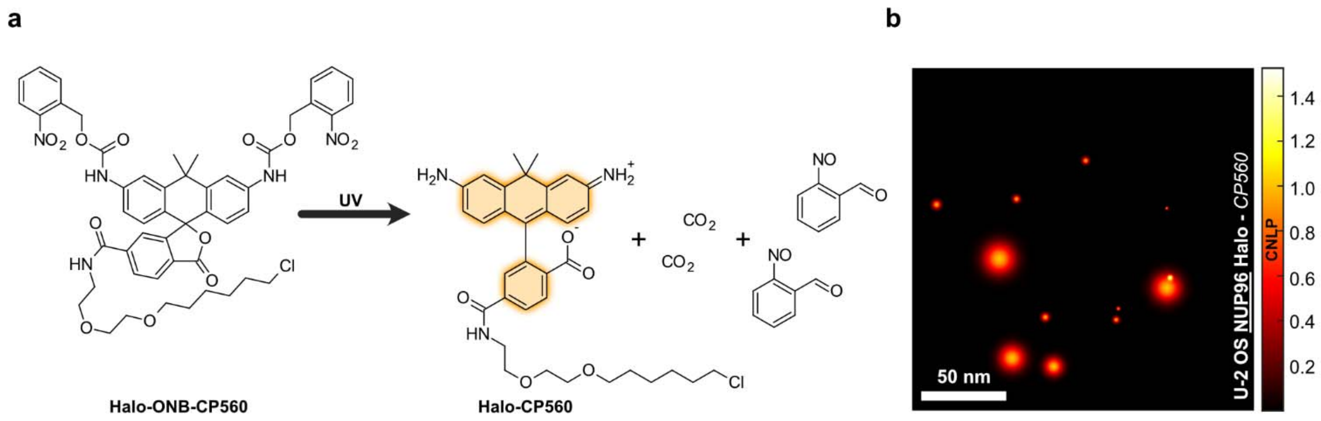

Suppl. Fig. S5 | Photoactivatable dye used in sample 5. a Activation follows irradiation with UV light. Synthesis will be published elsewhere. **b** NUP96 image from a U-2 OS cell recorded by Halo-tag labeling with the photoactivatable fluorophore Halo-ONB-CP560. While this labeling does not attain the same level of completeness as DNA hybridization, the image highlights the viability of blue-shifted MINSTED with photoactivatable fluorophores offering just a single on-off cycle.

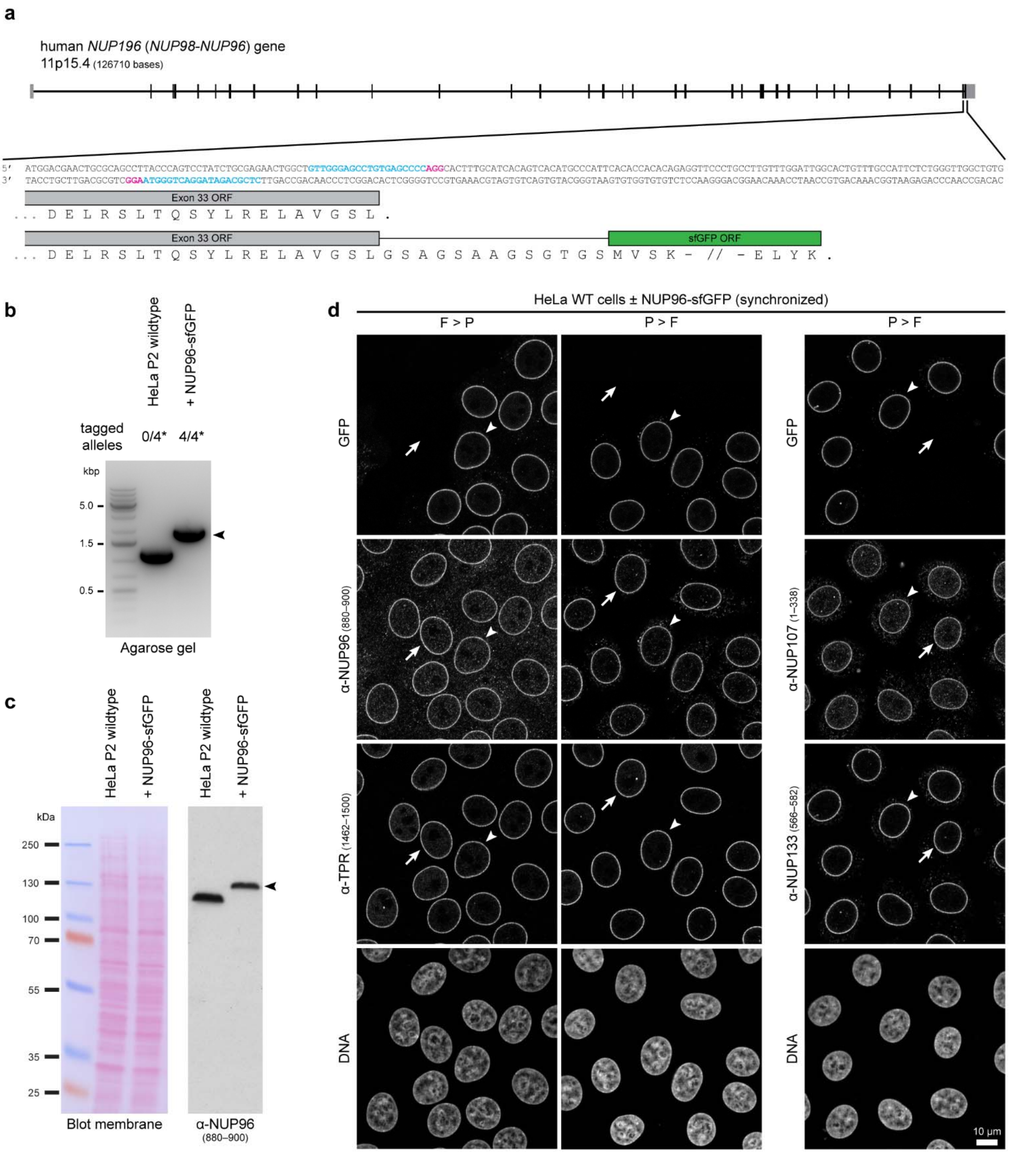

Suppl. Fig. S6 | Characterization of a HeLa cell line homozygously expressing sfGFP-tagged NUP96.

a Schematic depiction of the canonical human *NUP98-NUP96* gene, also known as *NUP196*, located on chromosome 11 at position 11p15.4. Among the gene's transcripts are such that encode for precursor polypeptides of 187 kDa to 199 kDa. These then undergo autoproteolysis[4], resulting in NUP98 as the amino-terminal polypeptide and NUP96 as the carboxy-terminal one. Nucleotide sequences presented correspond to part of the WT progenitor cells' last coding exon, which was the target for the insertion of the sfGFP ORF together with a flexible linker of 13 amino acids at the end of the NUP98-NUP96 ORF. Tagging by CRISPR/Cas9n methodology was in the HeLa P2 cell line. The pair of sgRNA sequences used for site-specific knock-in are shown in blue lettering except for each of the PAM sequences highlighted in magenta. In addition, the deduced aa sequences of the non-tagged WT progenitor and the tagged progeny cell line at the transitions between NUP96 and sfGFP-tag are provided for comparison. **b** Genotyping of the on-target integration of the sfGFP ORF via PCR amplification of genomic regions, with up to four *NUP98-NUP96* alleles (4*) present in the HeLa cell line P2, in accord with 11p15.4 occurring in four copies in other

lines of HeLa too[5, 6]. The PCR products, shown after agarose gel electrophoresis, stemmed from the original, tag-less (0/4*) progenitor cell line and the homozygous cell line with all *NUP98-NUP96* alleles tagged (4/4*). The arrowhead points at the PCR product, including the 717 bp-long sfGFP ORF. **c** Immunoblotting of total cell extracts from the tag-less HeLa progenitor cells and the progeny line with all *NUP98-NUP96* alleles tagged. Labelling for NUP96 with an antibody targeting an epitope (aa 880–900) located near the protein's carboxy-terminus was performed on the membrane shown stained with Ponceaus S on the left. The arrowhead points at the band that represents the sfGFP-tagged NUP96 polypeptides. **d** Immunofluorescence microscopy of HeLa P2 progenitor cells grown together with the NUP96-sfGFP-expressing progeny cells as mixed populations on the same coverslips. Cells had been cell cycle-synchronized and then double-immunolabelled with combinations of antibodies for NUP96, other Y-complex antibodies like NUP107 and NUP133, and the Y-complex-attached nuclear basket scaffold protein TPR. Furthermore, cells had been chemically fixed before their permeabilization with detergents (F > P) as well as after having been permeabilized first (P > F). Arrows mark some of the NUP96-sfGFP-expressing cells' NEs, while arrowheads mark the NEs of some neighbouring progenitor cells with the tag-less NUP96 polypeptides. Note that immuno-labelling intensities at the NEs of the NUP96-sfGFP cells appear very similar to those in the progenitor line, irrespective of whether cells had been permeabilized before or after fixation, as here exemplified for NUP96 and TPR. This indicates that NUP96-sfGFP copy numbers incorporated into the NPCs remain similar to NUP96 copy numbers incorporated into the progenitor cells' NPCs. Moreover, these findings demonstrate that the sfGFP tag and the linker by which the tag is appended to NUP96 neither notably affects NPC incorporation of other Y-complex proteins nor the nuclear basket's attachment to the NPC. Bar, 10 μm. Rabbit polyclonal anti-hsNUP107 (1-338) (19217-1-AP, Proteintech), rabbit polyclonal anti-hsNUP96 (880-900)[7] ,mouse monoclonal anti-hsTPR, clone 203-37 (1462-1500)[8, 9] and guinea pig polyclonal anti-hsNUP133 (566-582)[10] were used.

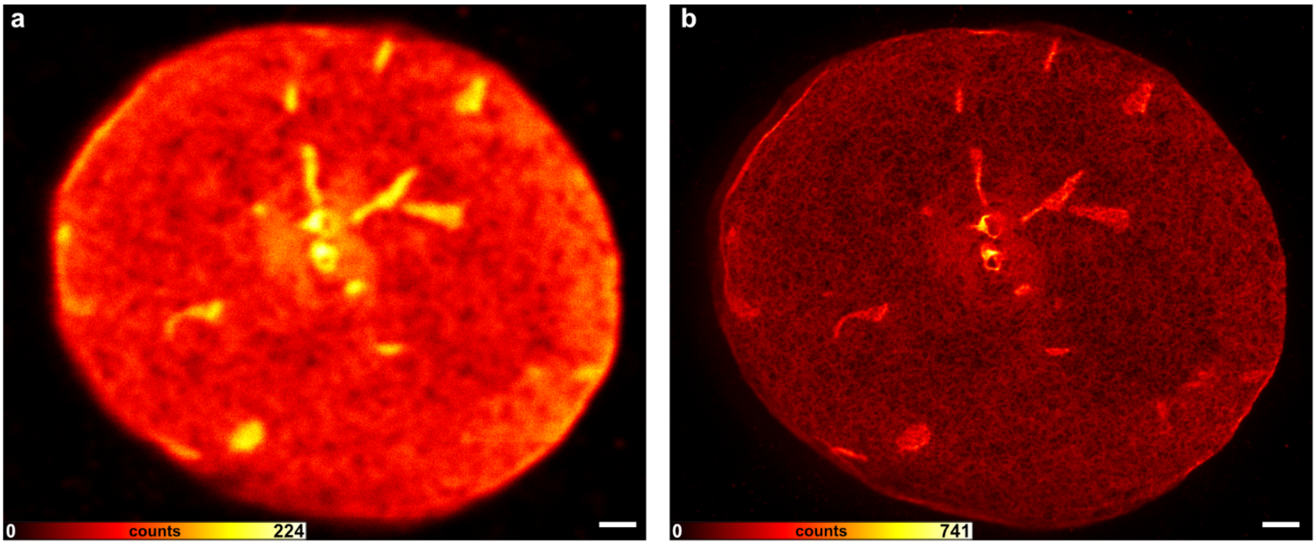

Suppl. Fig. S7 | COS-7 cells with lamin A/C labelled. To investigate the lamin A/C primary antibody specificity, COS-7 cells were prepared analogous to sample 6, but with a secondary antibody carrying a STAR RED fluorophore for direct STED imaging. Samples were imaged on an Abberior Instruments expert line. **a** Confocal imaging. **b** STED imaging with a STED wavelength of 775 nm. Scalebar 1 µm.

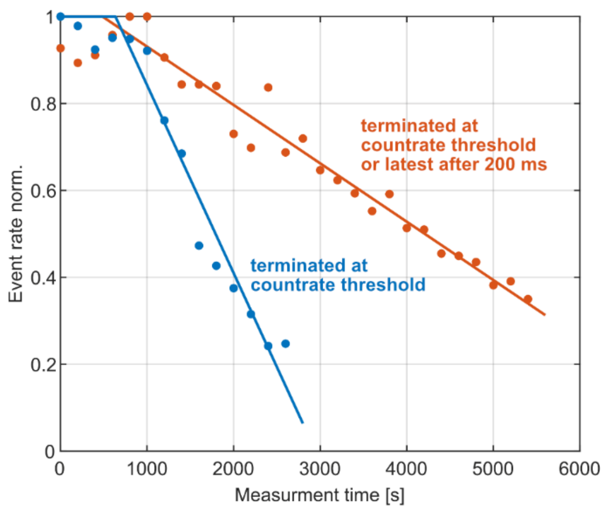

Suppl. Fig. S8 | MINSTED DNA PAINT binding site bleaching. The graph shows how terminating localizations after a maximum duration of 200 ms effects the event rate. From this, we conclude that binding site bleaching is strongly reduced if localizations are terminated early. The red dots refer to the data from Fig. 6a (sample 6) and the blue dots to a measurement on an analogously prepared sample. The lines are linear fits with a saturation at a value of 1.

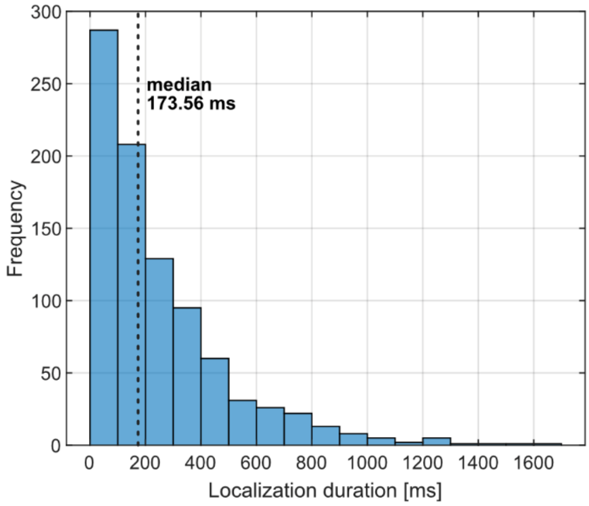

Suppl. Fig. S9 | Distribution of localization duration. The duration of localizations during the measurement presented in Fig. 4a (sample 1) is histogrammed.

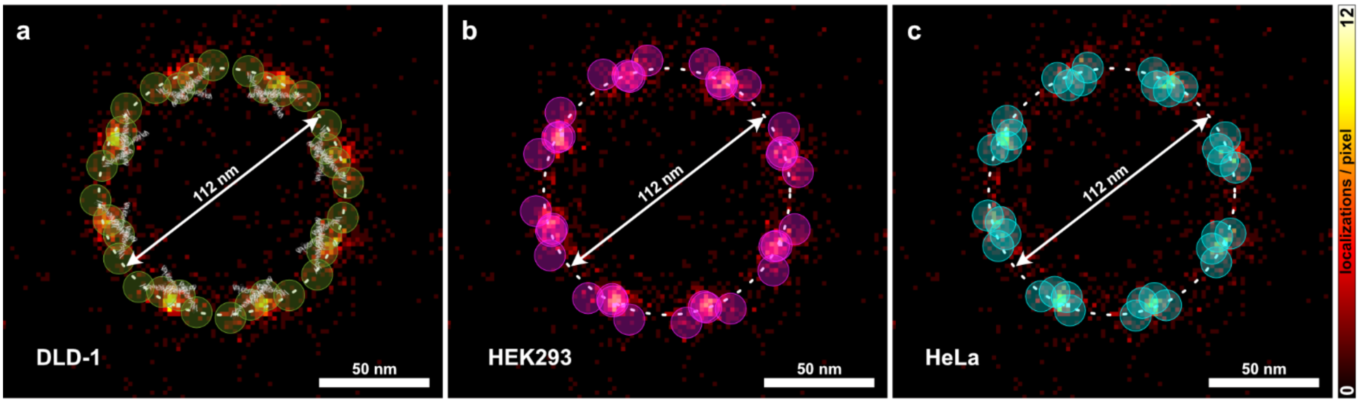

Suppl. Fig. S10 | MINSTED overlay combined with cryo-ET data. The subfigures each show a histogram of the overlaid 81 MINSTED images relative to the full-length structures of the NUP96 polypeptides or their C-termini. In addition, the MINSTED data's computed mean diameter of 112 nm is outlined. Three cryo-ET datasets used for comparison comprise the cryo-milled NPCs of **a** DLD-1 and **b** HEK293 cells, and **c** the isolated NPCs of HeLa cells. From the cryo-ET datasets, we infer the possible 2D projected fluorophore positions as circular areas with a radius of 7 nm, as an estimated distance between NUP96 C-terminus and fluorophore, around the C-termini of the NPC's 32 NUP96 polypeptides.

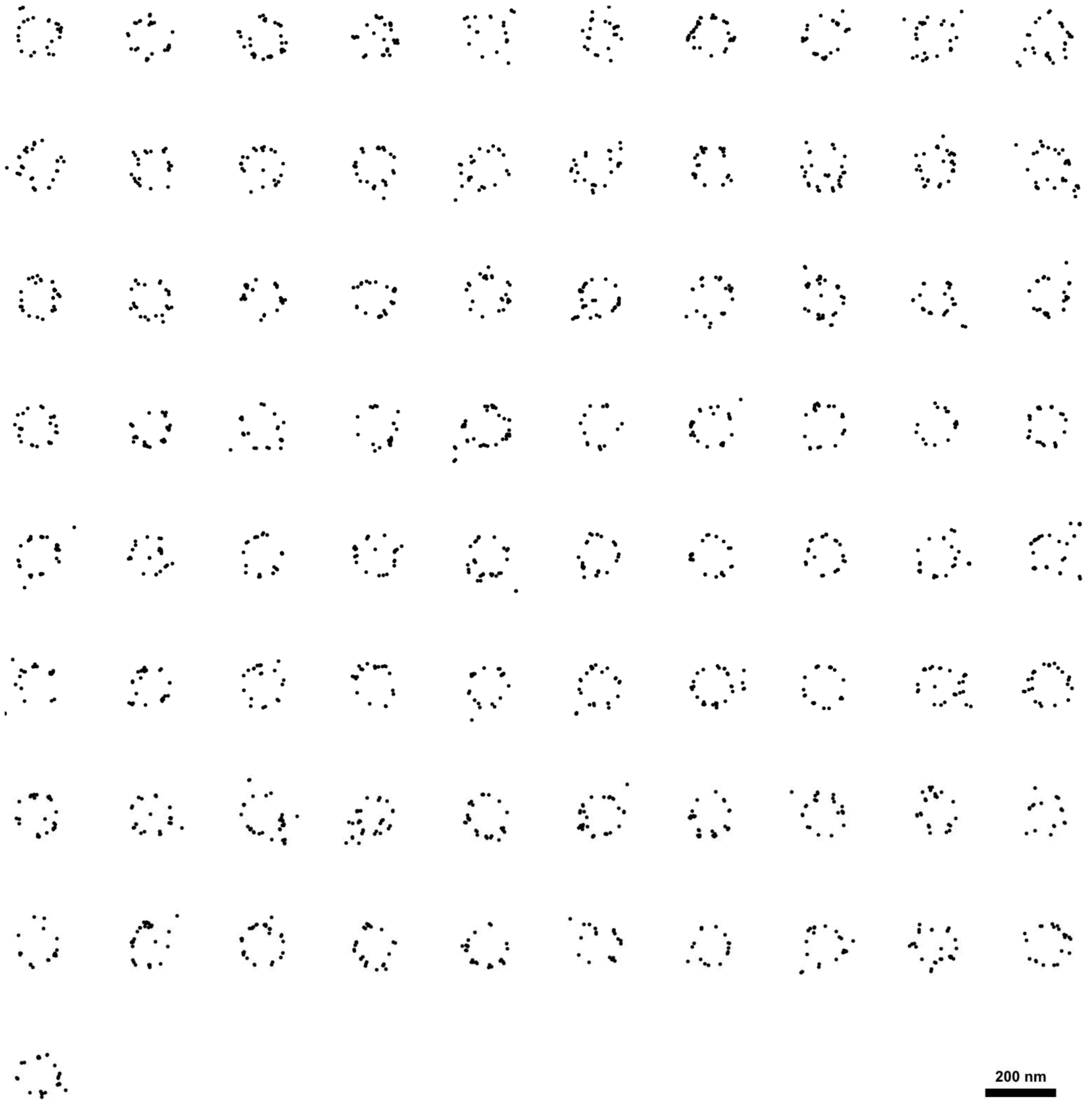

Suppl. Fig. S11 | Single nuclear pore images. Scatter plots of the 81 MINSTED nuclear pore images, which were used in order to compute the mean diameter as well as to derive the particle averaged structure, shown in Fig. 5h. From all initially selected nuclear pore images, the ones presented above were analysed to have eight occupied sites in the eightfold arrangement and showed an ellipticity of < 1.25. These filtering parameters were applied to ensure a dataset of nuclear pore images with a good coverage along the outline of the pore and to exclude tilted ones to support a good estimate of the pore diameters.

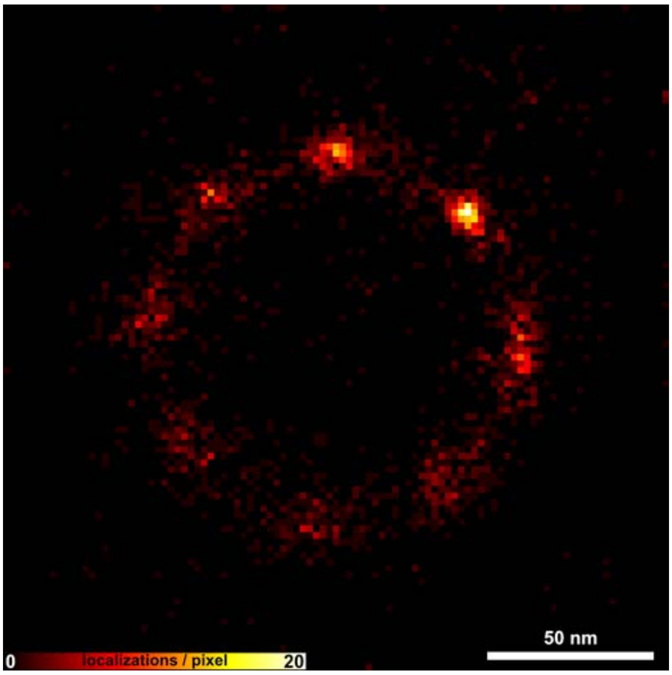

Suppl. Fig. S12 | Initial overlay image. Histogram of the initial particle averaging result. In this, the eightfold symmetry is well visible, while one of the clusters is strongly signal-enhanced. This is caused by the objective function of the particle averaging algorithm, which prefers to map each particle's site of highest density onto the global maximum of the overlay. In order to compensate for that, each of the 81 particles was rotated by a random multiple of 45°, followed by another round of particle alignment, which led to the final image as shown in Fig. 5h.

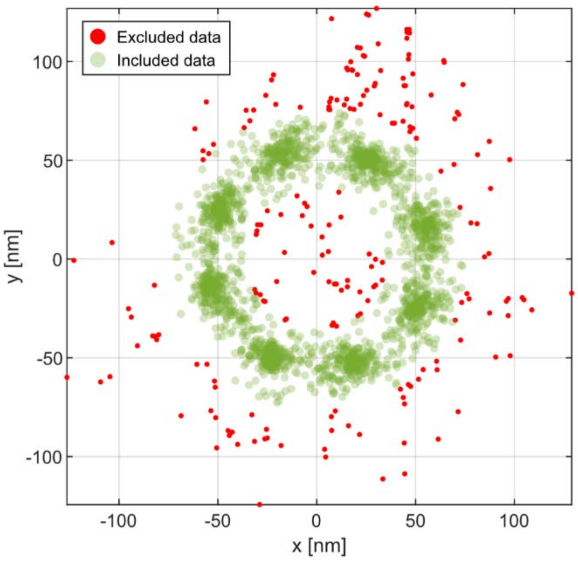

Suppl. Fig. S13 | Filtering of localizations for EM-MINSTED overlay. In order to estimate the fraction of localizations included within the vicinity of the NUP96 C-termini, with respect to their distance between (Fig. 5i), localizations with a radial distance of > 20 nm from the mean diameter were excluded from the dataset (8.6% excluded). Some of such localizations probably arose from perturbing binding sites of neighboring pores, unspecific binding of the nanobody or DNA strand.
